# Integrated analysis of transcriptomics and metabolomics and high-throughput amplicon sequencing reveals the synergistic effects of secondary metabolites and rhizosphere microbiota on root rot resistance in *Psammosilene tunicoides*

**DOI:** 10.3389/fmicb.2025.1554406

**Published:** 2025-04-14

**Authors:** Li Yingtao, Li Qiaofeng, Wang Lijuan, Qi Shuyun, Jiang Zhou, Zhang Wenping, Zhang Aili

**Affiliations:** ^1^Key Laboratory of Sustainable Utilization of Southern Medicinal Resources in Yunnan Province, Yunnan University of Chinese Medicine, Kunming, China; ^2^School of Traditional Chinese Medicine, Yunnan University of Chinese Medicine, Kunming, China

**Keywords:** root rot, *Psammosilene tunicoides*, multi-omics, “plant-microbe” interaction network, plant defense mechanisms, root microbiome

## Abstract

*Psammosilene tunicoides* is a plant with significant medicinal and ecological value, exhibiting remarkable medicinal properties, particularly in anti-inflammatory, antioxidant, and immune-regulatory effects. Root rot is one of the primary diseases affecting *Psammosilene tunicoides*, leading to a significant decline in its quality. In this study, we utilized an integrated analysis of transcriptomics, metabolomics, high-throughput amplicon sequencing, and culturomics for revealing the difference of healthy samples (CH) and diseased samples (CD) and studying the defense mechanism of *P. tunicoides* in resisting root rot. Transcriptome revealed distinct patterns of gene expression between healthy root samples (HR) and diseased root samples (DR) of *P. tunicoides. The* Key enzyme genes involved in triterpene (e.g., *HMGS*, *DXS*, *SQS*, *CYP450*) and flavonoid (e.g., *PAL*, *CHS*, *CHI*) biosynthesis pathways were significantly upregulated in DR. Consistent results were observed in the metabolomic analysis, where triterpene saponins and flavonoids were more highly accumulated in DR than in HR. Microbiome data indicated a significant enrichment of Actinobacteria at the genus level in the rhizosphere soil of diseased samples (DS) compared to healthy samples (HS) while the mostly beneficial growth-promoting bacterial groups were found in DR root endophytes, including *Enterobacter*, *Pseudomonas*, *Klebsiella*, *Stenotrophomonas*, and *Bacillus.* Through culturomics, we successfully isolated and identified over 220 bacterial strains from the rhizosphere soil of diseased samples, including genera including *Bacillus*, *Streptomyces*, *Cupriavidus*, *Pseudomonas*, and *Paenarthrobacter.* Notably, the strain *Pseudomonas* sp., which was significantly enriched in DR, exhibited a clear antagonistic effect against *Fusarium oxysporum*. Co-occurrence network analysis of multi-omics data revealed that many *Actinomycetes* positively correlated with triterpenoid and flavonoid compounds and their key genes. Therefore, we conclude that these secondary metabolites may could resist pathogen invasion directly or serve as an “intermediate medium” to recruit growth-promoting microorganisms to resistant the root rot. This study investigates the “Plant-Microbe” interaction network associated with root rot resistance in *P. tunicoides*, revealing its significant implications for the ecological cultivation and management of this species.

## 1 Introduction

*Psammosilene tunicoides* is a perennial herbaceous plant that only grows in Yunnan, Sichuan, Guizhou, and Tibet Provinces in China. This highly valued traditional Chinese medicine possesses analgesic, anti-inflammatory and immunoregulatory properties, and it is widely used in Chinese patented medicine, such as “Yunnan Baiyao,” a well-known traditional Chinese brand of herbal medicine for bone injuries (Zhang et al., 2010; Li et al., 2023). *P. tunicoides* has been utilized for hundreds of years in its wild form. However, its supply has gradually declined in recent years because of over-harvesting, and it no longer meets the commercial demand. In the process of *P. tunicoides* cultivation, root rot can cause wilting in the roots, leading to plant death (Li et al., 2024). Root rot diseases continue to pose a significant global threat to the productivity, quality, and marketability of medicinal herbs in China. These diseases are typically caused by multiple pathogens and are therefore often referred to as root rot complexes. Fungal and oomycete species are the predominant causative agents, while bacteria and viruses also contribute to root rot development (Williamson-Benavides and Dhingra, 2021).

The interaction between plants and their rhizosphere microorganisms plays a crucial role in plant growth and disease resistance. Plants have evolved to produce a vast array of specialized metabolites. This metabolic diversification was likely driven by the need to adapt to different environmental niches and by defense signaling under biotic and abiotic stresses (Cook et al., 1995). Specifically, plant roots can release a variety of root exudates, such as sugars, amino acids, organic acids, enzymes and second metabolites. Firstly, these root exudates, including secondary metabolites, Then, these root exudates can attract beneficial microbes, leading to the formation of a complex microbial community (Weller, 1998). Moreover, root exudates can also serve as signaling molecules, facilitating communication between plants and microorganisms and thereby regulating microbial growth, metabolism and functionality (Li et al., 2021). However, the mechanisms by which *P. tunicoides* interacts with microbes to defend against plant diseases are not well understood. A previous study reported that gene transcription regulates excretion by plant roots, which shapes the root-associated microbial community and the ability to resist root rot (Pang et al., 2022). We hypothesized that diseased root may change the *P. tunicoides* gene transcription, metabolic processes and belowground bacterial and fungal communities.

Recent technological advances have transformed plant disease ecology, allowing researchers to adopt a holistic framework for studying pathogenesis and its underlying mechanisms. With technological advancements, combined analysis of the plant transcriptome and metabolome and amplicon sequencing have improved our understanding of the interactions between the plant microbiome and hosts, thus promoting the development of agriculture, plant resistance and performance and other aspects (Priya et al., 2023). To explore the effects of root rot on *P. tunicoides* gene transcription, functional metabolites or the belowground microbiota, we conducted combined transcriptome, metabolome and high-throughput amplicon sequencing in healthy and diseased *P. tunicoides* root samples and rhizosphere soil samples to reveal the potential mechanisms, defense systems and root rot pathogens. Therefore, this study aimed to reveal the characteristics and potential pathogenic mechanism of root rot in *P. tunicoides*, explore the mechanism by which “plant–microbe” interactions prevent root rot in *P. tunicoides* and identify potential resistance genes and metabolites for microbiota biocontrol.

## 2 Materials and methods

### 2.1 Collection of plant and soil samples

In 2021, six healthy (CH) and six diseased (CD) *P. tunicoides* plant samples were randomly collected from a single site in a village (Yuhua Village) within Diannan Town, Jianchuan County, Dali City, Yunnan Province, China (26.24° N, 99.00° E). Jianchuan County is the place in Yunnan Province where the largest area of *P. tunicoides* is planted. In our study, all the collected *P. tunicoides* samples were cultivated for 1 year, exhibiting consistent growth. In addition, the plants were cultivated in a semi-natural environment with minimal human intervention, which was limited to weeding without fertilization. Then the plant samples were identified by Professor Qian Zigang of Yunnan University of Traditional Chinese Medicine.

Six biological replicates for each category were randomly collected. Among them, three biological replicates of the root of *P. tunicoides* were used for both transcriptomic and metabolomic analyzes. For healthy root (HR) and diseased root (DR) collection, the roots were surface-sterilized with 75% ethanol for 1 min, followed by treatment with 1% sodium hypochlorite for 30 s. The roots were rinsed six times with sterile water, rapidly frozen in liquid nitrogen, transported in dry ice to the laboratory and stored at −80°C. These samples were used for subsequently used for transcriptomic and metabolomic studies.

Six biological replicates of roots and rhizosphere soil were used for sequencing of the 16S rRNA gene amplicon sequencing and internally transcribed spacer (ITS) regions. The methods of the root collection were mentioned above. To collect rhizosphere soil, the roots were vigorously shaken to remove loose soil. The soil adhering to the roots (1–2 mm thick) was gently brushed into sterile centrifuge tubes and used as the rhizosphere soil. Rhizosphere soil samples from healthy (HS) and diseased (DS) specimens were processed independently. One portion of each sample was utilized for the isolation of culturable bacterial strains, while the other portion was subjected to 16S ribosomal RNA and ITS sequencing. For both sequencing analyzes, six biological replicates were prepared for each rhizosphere soil sample. RNA Extraction and transcriptomic Analysis of Healthy and Diseased *P. tunicoides* Roots.

Total RNA was extracted using Trizol reagent (Thermo Fisher Scientific, Waltham, MA, United States) following the manufacturer’s procedure. RNA quantity and purity were assessed using a Bioanalyzer 2100 (Agilent, Santa Clara, CA, United States) and RNA 1000 Nano Labchip Kit (Agilent), with RNA integrity number > 7.0 denoting sufficient integrity. Poly (A) RNA was purified from 5 μg of total RNA using poly-T oligo-attached magnetic beads with two rounds of purification. The mRNA was then fragmented using divalent cations under elevated temperature. The cleaved RNA fragments were reverse-transcribed to generate the final cDNA library according to the mRNASeq sample preparation kit (Illumina, San Diego, CA, United States) with an average insert size of 300 ± 50 bp. Paired-end sequencing was performed on a Novaseq™ 6000 (Illumina) at LC Sciences (Houston, TX, United States) following the vendor’s protocol.

For bioinformatics analysis, Cutadapt and in-house Perl scripts were used to remove reads with adaptor contamination, low-quality bases and undetermined bases. Sequence quality was verified using FastQC, including Q20, Q30 and GC-content metrics. *De novo* assembly of the transcriptome was performed using Trinity 2.4.0, which groups transcripts into clusters based on shared sequence content. The longest transcript in each cluster was chosen as the unigene sequence. Assembled unigenes were aligned against the non-redundant protein database and the Gene Ontology (GO), SwissProt, Kyoto Encyclopedia of Genes and Genomes (KEGG) and eggNOG databases using DIAMOND with an E-value threshold of <0.00001. Differential expression analysis was conducted using Salmon to calculate the transcripts per million values, and differentially expressed unigenes were identified by log2 fold change |Log2FC| > 1 or Log2FC < −1 and statistical significance (*p* < 0.05) using the R package edgeR.^1^ The KEGG^2^ was used for metabolic pathway analysis, while KOBAS software was used for the functional enrichment analysis (adjusted *p* < 0.05). MapMan version 4.6.0 RC1 was used for visualizing differentially expressed unigenes mapped to various pathways or processes.

### 2.2 Comparative metabolomic analysis of healthy and diseased *Psammosilene tunicoides*

Biological samples were vacuum freeze-dried using a lyophiliser (Scientz-100F, Scientz Instruments Co., Ltd., Suzhou, China). The samples were then ground to a powder using a grinder (MM 400, Retsch, Haan, Germany) at 30 Hz for 1.5 min. Fifty milligrams of the powdered sample were weighed using an electronic balance (MS105DΜ), and 1,200 μL of −20°C pre-cooled 70% methanolic aqueous internal standard extract was added (1,200 μL of extractant per 50 mg of the sample). The sample was vortexed six times (30 s every 30 min) at 4°C. After centrifugation at 12,000 rpm for 3 min at 4°C, the supernatant was aspirated, filtered through a microporous membrane (0.22 μm pore size), and stored in an injection vial for ultra-performance liquid chromatography–tandem mass spectrometry (UPLC-MS/MS) at −20°C.

The sample extracts were analyzed using a UPLC-electron spray ionization (ESI)-MS/MS system (UPLC, ExionLC^™^ AD, Sciex, Framingham, MA, United States; MS, QTRAP 4500, Sciex). Concerning the analytical conditions, the UPLC column was an SB-C18 column (1.8 μm, 2.1 mm × 100 mm, Agilent), and the mobile phase consisted of solvent A (pure water with 0.1% formic acid) and solvent B (acetonitrile with 0.1% formic acid). Sample measurements were performed with a gradient program starting at 95% A and 5% B. A linear gradient was applied over 9 min to 5% A and 95% B, which was maintained for 1 min. Then, the composition was adjusted to 95% A and 5% B within 1.1 min and held for 2.9 min. The flow rate was set at 0.35 mL/min. The column oven temperature was 40°C, and the injection volume was 4 μL. The effluent was connected to an ESI-triple quadrupole-linear ion trap MS system.

The ESI source parameters were as follows: source temperature, 550°C; ion spray voltage, 5,500 V (positive ion mode)/−4,500 V (negative ion mode); ion source gasses I and II and curtain gas were set at 50, 60 and 25 psi, respectively; and collision-activated dissociation was set to high. QQQ scans were acquired as multiple reaction monitoring (MRM) experiments with collision gas (nitrogen) set to medium. Declustering potential and collision energy for individual MRM transitions were further optimized. A specific set of MRM transitions was monitored for each period according to the metabolites eluted within that period.

Unsupervised principal component analysis (PCA) was performed using the prcomp function in R. The data were unit variance-scaled before PCA. The hierarchical cluster analysis (HCA) results for samples and metabolites were presented as heatmaps with dendrograms, and Pearson’s correlation coefficient between samples was calculated using the core function in R and presented as heatmaps. Both HCA and Pearson’s correlation analyzes were conducted using the R package ComplexHeatmap. For HCA, the normalized signal intensities of metabolites (unit variance scaling) were visualized as a color spectrum. For two-group analysis, differential metabolites were identified according to variable importance in projection (VIP) > 1 and absolute Log2FC (|Log2FC| ≥ 1.0). To ensure the accuracy of the identified differential metabolites, statistical significance was assessed using Student’s *t*-test for two-group comparisons, and *Kruskal-Wallis* test for multi-group comparisons. The *p-values* were adjusted using the Benjamini-Hochberg method to control the false discovery rate. A significance threshold of *p* < 0.05 was applied. VIP values were extracted from the orthogonal partial least squares discriminant analysis (OPLS-DA) results, which also included score plots and permutation plots generated using the R package MetaboAnalystR. The data were log-transformed (log2) and mean-centered before OPLS-DA. To avoid overfitting, a permutation test (200 permutations) was performed, and the results were corrected using the Benjamini-Hochberg method. The identified metabolites were annotated using the KEGG Compound database,^3^ and the annotated metabolites were then mapped to the KEGG pathway database.^4^ Pathways with significantly regulated metabolites were subjected to metabolite set enrichment analysis, with significance determined by *p*-values generated using the hypergeometric test, and *p*-values were further adjusted using the Benjamini-Hochberg method to control false positives.

90-bp paired-end reads. Sequence data were processed with QIIME2 for demultiplexing, quality filtering and merging, and chimeric sequences were removed using VSEARCH. Operational taxonomic units (OTUs) were clustered at 97% similarity with UCLUST, and taxonomic classification was performed using the SILVA database for 16S rDNA and the UNITE database for ITS2. Diversity indices and statistical analyzes, including principal coordinate analysis (PCoA) and permutational multivariate analysis of variance, were conducted. Negative and positive controls were included to ensure data accuracy, and data integrity was verified by quality scores and read length distribution, with samples with fewer than 10,000 high-quality reads being re-sequenced or excluded. This protocol ensured reliable and reproducible microbial community characterization.

### 2.3 Isolation, identification and inoculation of pathogens and biocontrol bacteria

Initially, plant samples were immersed in 5% sodium hypochlorite solution for 5 min and rinsed with 2.5% sodium thiosulfate solution for 10 min to remove residual sodium hypochlorite. The samples were then soaked in 75% ethanol for 3 min and rinsed three times with sterile water to remove ethanol residues. The final rinse was spread on a pre-prepared isolation medium and incubated at 28°C for at least 3 weeks to assess microbial growth, ensuring complete disinfection. To isolate pd-01, samples were collected from the boundary between healthy and diseased tissues of diseased *P. tunicoides* for microbial isolation. Thin sections or crushed tissues were placed on sterile filter paper in Petri dishes and dried at 30°C in a sterile environment. After treatment, the samples were ground in liquid nitrogen, and particles were transferred onto an isolation medium using sterile toothpicks. Plates were incubated at 28°C for 5–12 days. To isolate culturable biocontrol bacteria from the rhizosphere soil of diseased plants, 1 g of rhizosphere soil was suspended in 9 mL of 0.1% tetrasodium diphosphate solution containing 1 g of gravel and shaken for 25 min at 200 rpm. Serial dilutions (10^−2^ to 10^−4^) were prepared and plated onto Petri dishes (9 cm diameter) containing 1/10 tryptic soy agar medium. Five replicated Petri dishes were prepared for each dilution. When colonies appeared, single colonies or edge colonies were isolated using a sterile inoculation loop, streaked onto fresh medium and further incubated at 37°C to promote colony growth. To isolate potential pathogenic fungi, fungi were purified according to their morphology and color, with hyphal tips selected for further purification until a pure culture was obtained. Pathogenic fungi were isolated on potato dextrose agar (PDA; 200 g potatoes, 20 g glucose, 15 g agar powder and 1,000 mL distilled water), and biocontrol bacteria were isolated on Luria–Bertani medium (10 g peptone, 5 g yeast extract, 10 g sodium chloride, 15 g agar and 1,000 mL distilled water). Fungal pathogens were amplified using the primers ITS1F (5′-CTTGGTCATTTAGAGGAAGTAA-3′) and ITS4R (5′-TCCTCCGCTTATTGATATGC-3′). Bacterial DNA was extracted using a Qiagen DNA extraction kit. The 16S rRNA gene was amplified using the primers 27F (5′-AGAGTTTGATCMTGGCTCAG-3′) and 1492R (5′-TACGGYTACCTTGTTACGACTT-3′) and sequenced by Qiagen Biotechnology Co. Sequences were compared to those in the NCBI ITS (fungal) and 16S rRNA (bacterial and archaeal) databases using BLAST to determine phylogenetic relationships, which were visualized using MEGA8 software, and phylogenetic trees were constructed using the neighbor-joining method.

To confirm pathogenicity, a spore suspension of *F. oxysporum* with a concentration of 1.0 × 10^7^ CFU/mL was prepared. The roots of 10-month-old *P. tunicoides* plants were immersed in the spore suspension for 30 min, after which they were planted in pots. For the control group, sterile water was used instead of the fungal suspension for inoculation. Both the control group and the fungal treatment group consisted of six replicates each. Typical wilting symptoms, consistent with those observed in naturally infected plants, were monitored following fungal inoculation. At this stage, pathogenic fungi isolated from diseased roots were subjected to ITS sequencing to validate Koch’s postulates. To evaluate the *in vitro* antagonistic activity of biocontrol bacteria isolates against pathogenic fungi, fungal mycelial disks (5 mm in diameter) were transferred to the center of newly prepared PDA plates. Biocontrol bacterial isolates were then inoculated 3 cm from the center of the pathogenic fungal colonies. Plates without pathogenic fungi served as controls. Each treatment was repeated six times. After incubation in the dark at 28°C for 7 days, antagonistic activity was assessed by measuring the growth diameter of the pathogenic fungi. Correlation Heatmap and Co-occurrence Network Analysis.

To comprehensively analyze the relationships between microbial genera, key genes, and metabolites, two distinct analytical approaches were employed: correlation heatmap analysis and co-occurrence network analysis. Both approaches were based on multi-omics quantitative data, but they explored the relationships within and between omic layers from different perspectives. In Correlation Heatmap analysis, we used R version 4.2.0 with the ggcor package (version 0.9.8.1) for data processing and to calculate correlations between microbial genera, key genes, and metabolites. The correlation matrix was assessed using Pearson correlation coefficients, and the heatmap visualized the intra-omics correlations. Strongly correlated omic pairs were connected with red lines, indicating significant differences (*p* < 0.01, adjusted using the Benjamini-Hochberg FDR method), while green lines represented weaker but still significant correlations (*p* < 0.05, adjusted using the Benjamini-Hochberg FDR method). The intensity of the colors in the heatmap reflected the strength of the correlations, providing a more intuitive display of the interrelationships between omic features. The statistical significance of correlations was determined using the Benjamini-Hochberg procedure to control for false discovery rates (FDR) in multiple comparisons, with a significance threshold set at FDR < 0.05.

Co-occurrence networks were constructed to further reveal the interactions between microbial communities, key genes, and metabolites. These networks were visualized using Gephi 9.2 (Gephi Consortium), and the number of nodes was evaluated. The network was built by filtering significant features (such as filtered microbial, key genes, and metabolites), and the correlations between them were determined using R version 4.2.0. Positive correlations were represented in red, while negative correlations were displayed in blue. The βNTI values were calculated for each sample pair, with|βNTI| > 2 indicating that the interactions were governed by deterministic processes, while|βNTI| < 2 suggested that the interactions were likely driven by stochastic processes. Statistical differences were determined using SPSS 26.0 (IBM Corp., Armonk, NY, United States), and Tukey’s multiple range test was used to check for significant differences between means (*p* < 0.05). The *p*-values were adjusted for multiple comparisons using the Bonferroni correction.

## 3 Results

### 3.1 Comparative transcriptome analysis of healthy and diseased *Psammosilene tunicoides*

**Figure 1:**
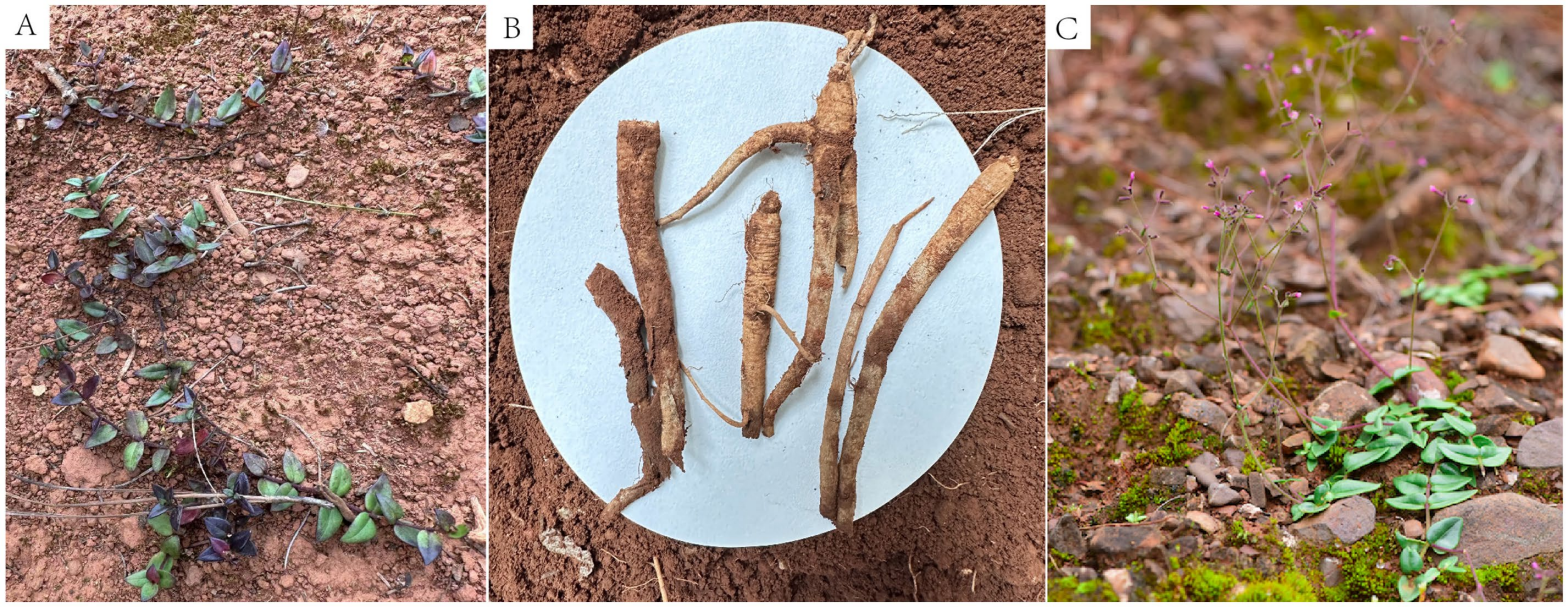
Comparison of healthy and root rot-affected *P. tunicoides* plants. **(A,C)** Healthy plants. **(B)** Root rot-affected plants.

**Figure 2:**
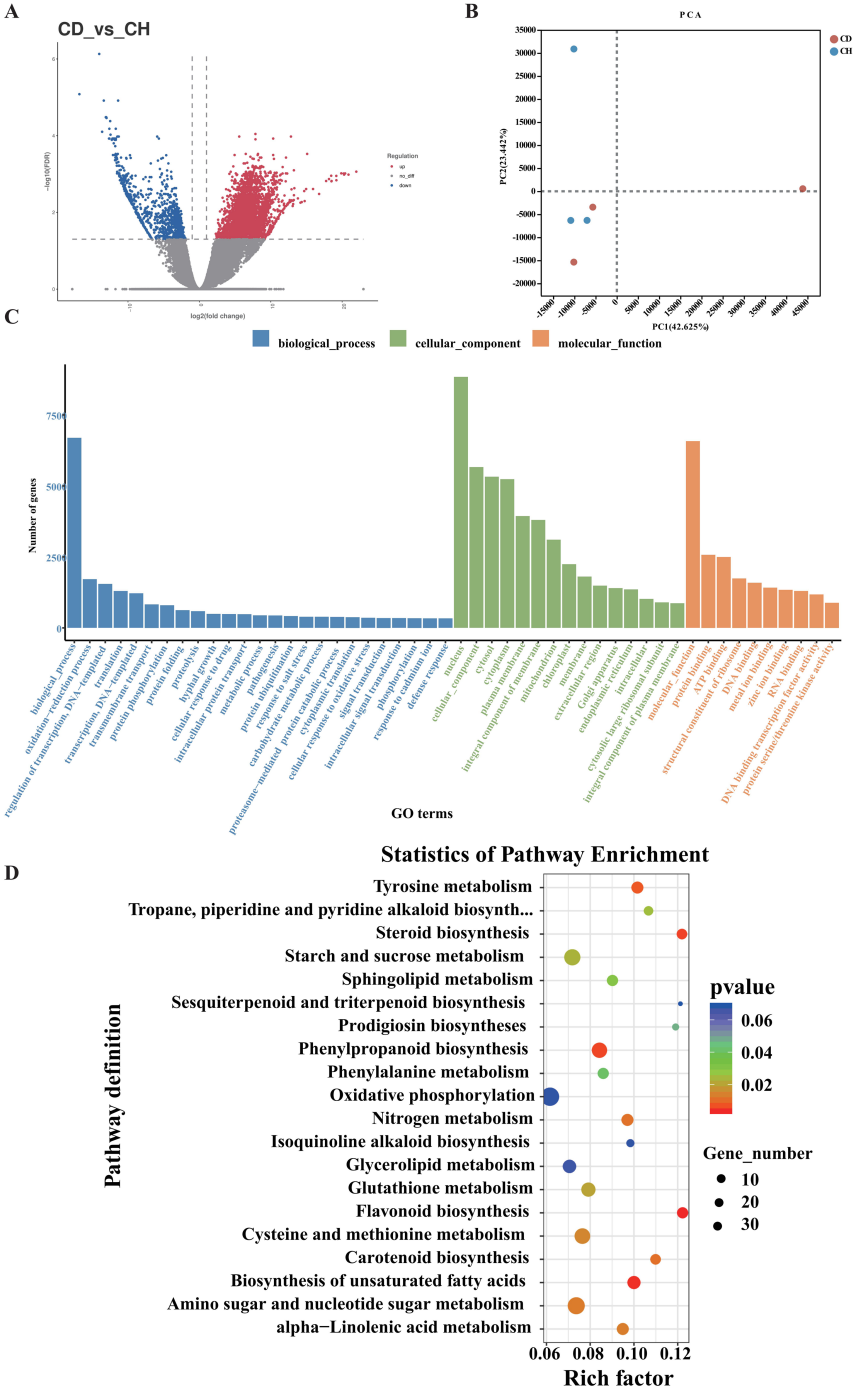
Functional enrichment analysis of DEGs in *P. tunicoides.* **(A)** Volcano plot of DEGs. **(B)** PCA score plot of the CD and CH groups. **(C)** GO enrichment histogram. **(D)** KEGG pathway enrichment scatter plot of DEGs.

Root rot changes *P. tunicoides* root metabolism and reduces the fresh weight. The diseased P. tunicoides displayed that leaves and stems withered and yellow, root rot, retarded growth, and plant death, and the yield was reduced (Figure 1). We sequenced six samples each from the CH and CD groups, generating 492,107,166 raw reads. After quality control, we obtained 324,970,148 clean reads, totalling 34.26 Gb of clean data. Each sample yielded more than 5.8 Gb of clean data with an error rate lower than 0.03%. Regarding quality indicators, Q20 exceeded 98%, Q30 exceeded 93%, and GC content ranged from 44.27 to 47.12% (Supplementary Table S1), confirming the reliability of our sequencing data. Among the identified unigenes, the longest and shortest were 11,885 and 201 bp, respectively (Supplementary Table S1). PCA revealed significant differences in principle components 1 and 2 (PC1 and PC2, respectively), indicating distinct gene expression patterns between the CH and CD groups. PC1 accounted for 42.625% of the variance, whereas PC2 explained 23.442% (Figure 2B).

We conducted differential expression analysis between the CD and CH groups using|Log2FC| > 1 and *q*-value < 0.05 as the criteria. As illustrated in Figure 2A, we identified 85,881 differentially expressed genes (DEGs), including 46,210 up-regulated and 35,785 down-regulated genes. These DEGs were categorized into three GO groups: biological process (GO: 0008150), cellular component (GO: 0005575) and molecular function (GO: 0003674; Figure 2C). Comparison of root rot-affected plants with healthy controls revealed significant enrichment of several GO terms across these categories. In the biological process category, 24 terms were enriched, most prominently “oxidation–reduction process,” “regulation of transcription,” “DNA-templated transcription,” “translation,” “transmembrane transport,” and “hyphal growth.” For cellular components, 14 terms were enriched, primarily “nucleus,” “plasma membrane,” and “mitochondrion and endoplasmic reticulum.” Nine terms were enriched in the molecular function category, with “binding” and “catalytic activity” being the major contributors (Figure 2C). These enriched terms are typically associated with plant responses to environmental stress and pathological conditions.

Additionally, KEGG pathway enrichment analysis of DEGs (Figure 2D) identified several significantly enriched pathways (Supplementary Table S2, *p* < 0.05) in diseased samples, specifically (in order of significance) flavonoid biosynthesis (map 00941), biosynthesis of unsaturated fatty acids (map01040), steroid biosynthesis (map00100), phenylpropanoid biosynthesis (map00940), tyrosine metabolism (map00350), sesquiterpenoid and triterpenoid biosynthesis (map00909), amino sugar and nucleotide sugar metabolism (map00520), cysteine and methionine metabolism (map00270) and starch and sucrose metabolism (map00500).

### 3.2 Comparative analysis of gene expression patterns in plant-pathogen interaction, triterpene saponin and flavonoid biosynthesis pathways between healthy and diseased *Psammosilene tunicoides*

KEGG enrichment analysis of the transcriptome (Supplementary Table S2) revealed significant enrichment of DEGs in secondary metabolite pathways, including “Plant hormone signal transduction,” “Flavonoid biosynthesis,” and “sesquiterpenoid and triterpenoid biosynthesis,” in diseased samples. Based on these findings, we focused on key enzyme genes involved in plant hormone signal transduction pathways (Supplementary Table S2), such as ABRE-binding factor (*ABF*), BRI1-associated receptor kinase (*BAK*), BR signaling-interacting protein, *BRI1* expressed, brassinazole-resistant (*BZR*), ethylene-insensitive (*EIN*), ethylene response factor (*ERF*), ethylene receptor (*ETR*), NAM/ATAF1/2/CUC2 (*NAC*), nonexpressor of pathogenesis-related gene 1 (*NPR1*), PYR-like receptor (*PYL*), pyrabactin resistance (*PYR*), TGACG motif-binding factor (*TGA*) and *WRKY* transcription factor (*WRKY*). Notably, the *ETR*, *ERF*, *NPR1*, *BAK*, and *WRKY* genes were consistently up-regulated in diseased samples, with most of the remaining genes also exhibiting up-regulation (Supplementary Figure S2; Supplementary Table S14).

**Figure 3:**
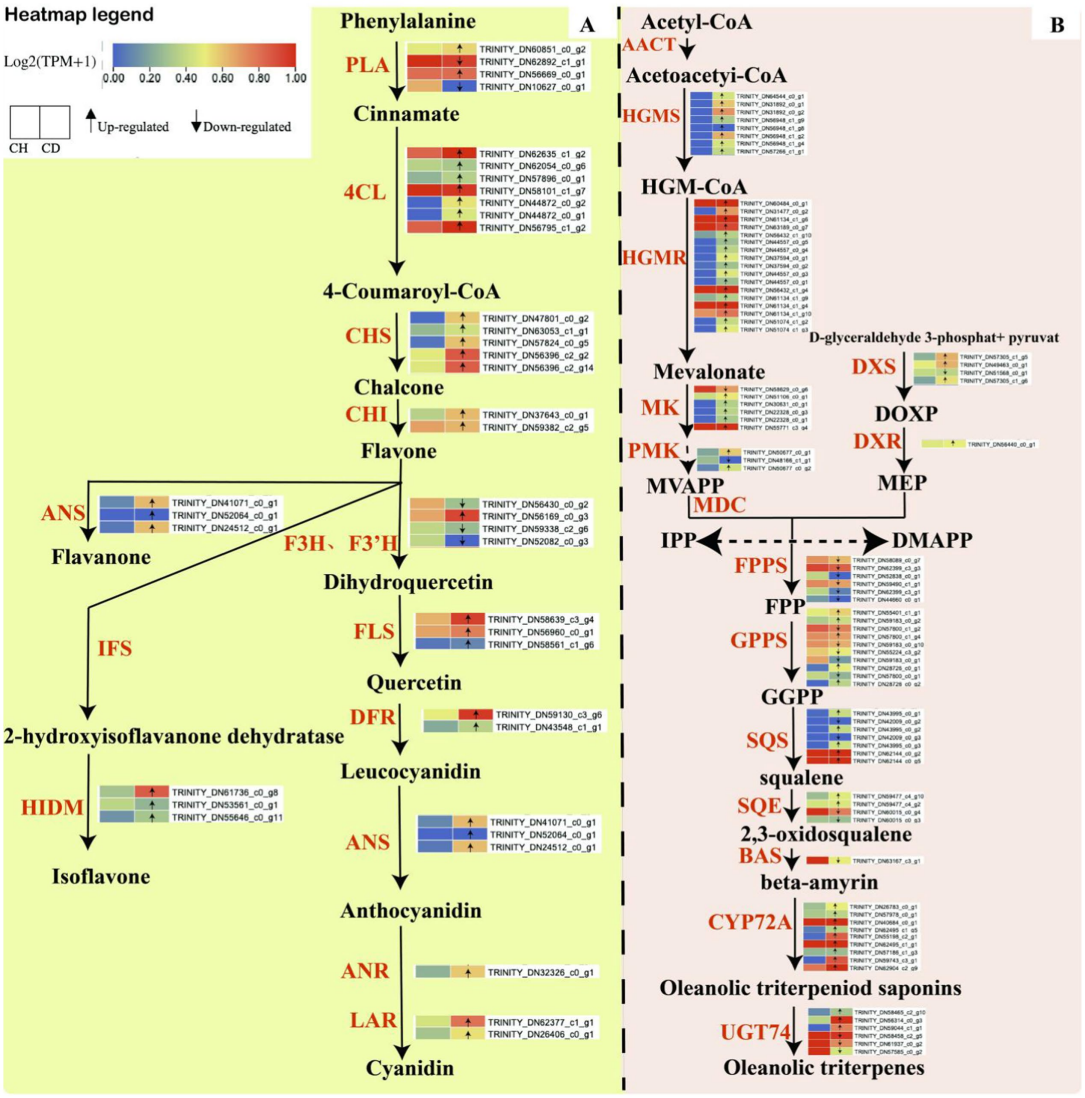
Biosynthesis pathways of flavonoid compounds and triterpene saponins in *P. tunicoides*. **(A)** Flavonoid biosynthesis pathway. **(B)** Triterpene saponin biosynthesis pathway.

We also examined key enzyme genes involved in the triterpene and flavonoid biosynthesis pathways to elucidate the accumulation patterns of triterpene saponins and flavonoids in healthy and diseased *P. tunicoides*. For triterpenes (Supplementary Table S3), we analyzed the genes hydroxymethylglutaryl-CoA synthase (*HMGS*), hydroxymethylglutaryl-CoA reductase (*HMGR*), mevalonate kinase (*MK*), phosphomevalonate kinase (*PMK*), geranyl diphosphate synthase (*GPPS*), farnesyl diphosphate synthase (*FPPS*), geranylgeranyl diphosphate synthase, squalene synthase (*SQS*), squalene epoxidase (*SQE*), beta-amyrin synthase (*bas*), UDP-glycosyltransferase (*UGT*), cytochrome P450 reductase (*CPR*), *UGT74* and *CYP72*. For flavonoids (Supplementary Table S4), we focused on the genes phenylalanine ammonia-lyase (*PAL*), 4-coumarate-CoA ligase (*4CL*), chalcone isomerase (*CHI*), naringenin 3-dioxygenase (*F3H*), flavonol synthase (*FLS*), dihydroflavonol 4-reductase (*DFR*), anthocyanidin synthase (*ANS*) and 2-hydroxyisoflavanone dehydratase (*HIDM*). Triterpene compounds are predominantly synthesized via the mevalonic acid (*MVA*) or methylerythritol phosphate (*MEP*) biosynthesis pathways (Vranová et al., 2013). Our transcriptome analysis identified genes encoding key enzymes for both pathways in healthy and diseased *P. tunicoides*, indicating that both pathways are active in this species (Figure 3A). We observed higher expression of *DXS* and *DXR* in the MEP pathway and higher expression of *HMGS* and *HMGR* in the *MVA* pathway in diseased *P. tunicoides* than in healthy samples. Moreover, DEGs involved in the synthesis of 2,3-oxidosqualene (a common precursor of triterpene saponins), such as *SQS* and *SQE*, exhibited elevated expression in diseased *P. tunicoides*. The *CYP72A* gene, crucial in the post-modification oxygenation stage of ginsenoside triterpene synthesis, was also up-regulated in diseased samples. Conversely, five *GPPS* genes, six *FPPS* genes and one *bas* gene were down-regulated in diseased *P. tunicoides* (Figure 3A).

Our analysis also identified genes encoding key enzymes in the main flavonoid compound pathway within the transcriptome data of healthy and diseased *P. tunicoides* (Figure 3B). The results revealed the up-regulation of most *PAL, 4CL*, chalcone synthase (*CHS*) and *CHI* genes in diseased *P. tunicoides*, leading to enhanced flavonoid accumulation. *ANS* genes, which are involved in flavanone synthesis, were up-regulated in diseased *P. tunicoides*, whereas *HIDM* genes, which participate in the final synthesis stage of isoflavones, exhibited higher expression in diseased plants (Wang et al., 2023). Furthermore, *FLS, DFR, ANS, LAR*, and *ANR* were significantly up-regulated in diseased *P. tunicoides*. These key enzyme genes catalyze the further production of various compounds from the flavonoid skeleton, including isoflavones and anthocyanins (Figure 3B).

### 3.3 Comparison of metabolite profiles between healthy and diseased *Psammosilene tunicoides*

To further investigate the dynamic changes in metabolites between root rot-affected and healthy *P. tunicoides*, we conducted metabolome analysis using widely targeted LC–MS/MS. The MS data were subjected to missing value imputation and low-mass ion removal (eliminating ions with more than 50% missing masses in quality control samples or more than 80% in actual samples), followed by data filtering. Subsequently, we collected statistical ion information. After data filtering, we identified 295 metabolites, which were categorized into 10 groups: flavonoids (25%), terpenoids (20%), lipids (14%), phenolic acids (11%), lignans and coumarins (5%), nucleotides and derivatives (4%), alkaloids (4%), organic acids (4%), amino acids and derivatives (3%), tannins (1%) and others (8%). The flavonoid compounds included flavones (41%), flavonols (24%), flavanols (12%), dihydroflavones (8%), isoflavones (8%), dihydroflavonols (3%), other flavonoids (3%) and chalcones (1%), and the terpenoid secondary metabolites comprised triterpenes (83%), sesquiterpenes (5%), triterpene saponins (5%), diterpenes (3%), and monoterpenes (3%; Supplementary Table S5).

**Figure 4:**
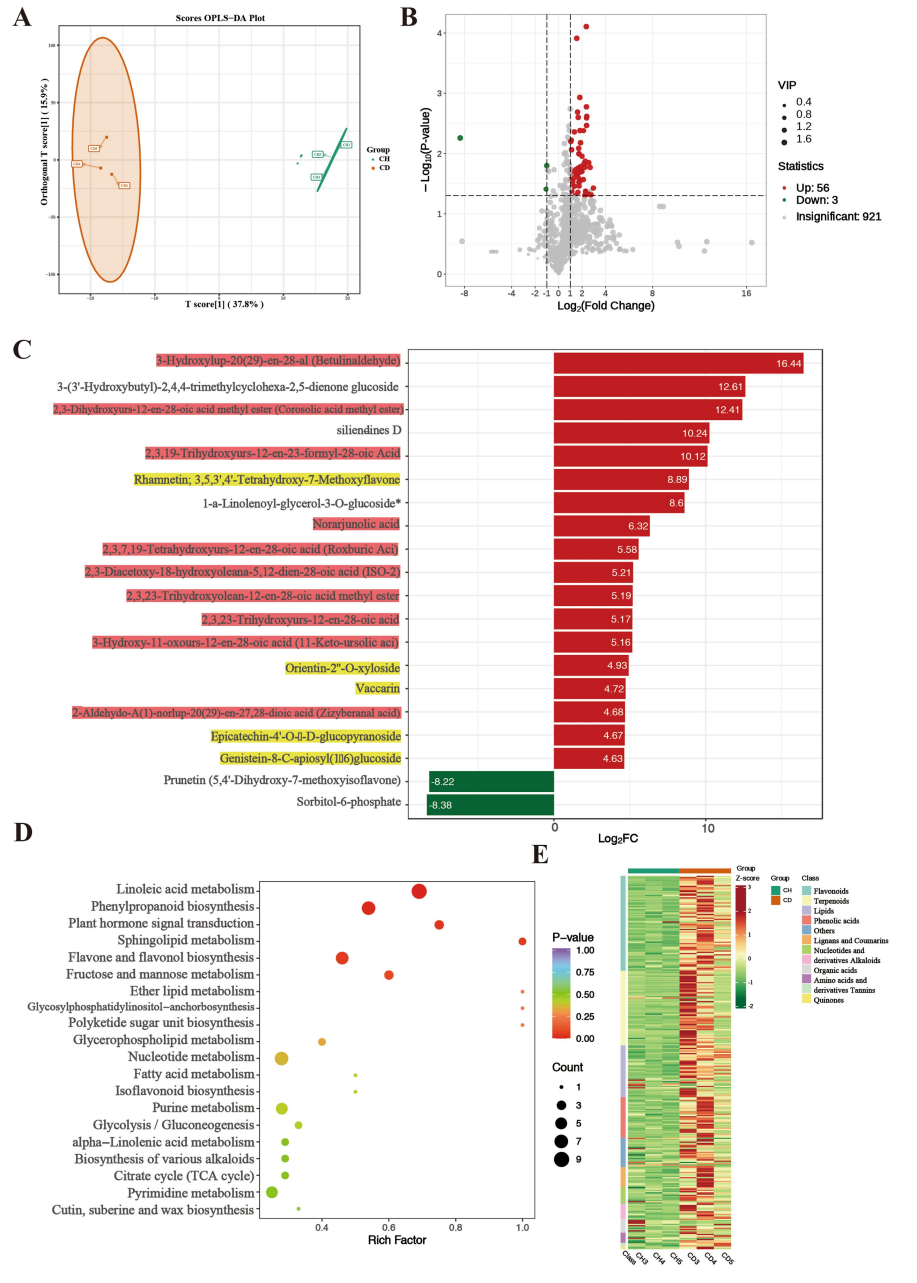
Comparative metabolomic analysis of healthy (CH) and diseased (CD) *P. tunicoides.* **(A)** OPLS-DA score plot of the CD and CH groups. **(B)** Volcano plot of DEGs in the CD and CH groups. **(C)** Expression analysis of top 20 significant DAMs (yellow: flavonoids, red: triterpenes). **(D)** KEGG pathways of DAMs. **(E)** Heatmap of metabolite classification.

We performed multivariate statistical analyzes using OPLS-DA. The OPLS-DA score chart revealed a clear separation trend between the two groups’ scores, with all samples falling within the 95% confidence interval (Figure 4A). This validated the samples for subsequent differential metabolite analysis. In this study, we filtered differentially accumulated metabolites (DAMs) from the total metabolites using the criteria of VIP ≥ 1, *p* < 0.05 and ratio ≥ 2 or ratio ≤ 1/2. The volcano plots identified 59 DAMs between healthy and diseased *P. tunicoides* (Figure 4B), including 56 and 3 metabolites with increased and decreased accumulation, respectively, in diseased samples. GO enrichment analysis (Figure 3C) of the top 20 significant DAMs illustrated that 18 DAMs were more strongly accumulated in diseased samples (Figure 4C, red for triterpenes, yellow for flavonoids). These included Ten triterpene compounds, namely 3,16-dihydroxy-23-oxoolean-12-en-28-oic acid (quillaic acid), norarjunolic acid, 3,24-dihydroxy-17,21-semiacetal-12(13) oleanolic fruit, 2,3-dihydroxyolean-12-en-28-oic acid (2-hydroxyoleanolic acid), 23-hydroxy-3-oxoolean-12-en-28-oic acid (hederagonic acid), 3,23-dihydroxy-30-noroleana-12,20(29)-dien-28-oic acid (30-norhederagenin), 3-oxoolean-12-en-28-oic acid (oleanonic acid), 2,3-diacetoxy-18-hydroxyoleana-5,12-dien-28-oic acid (ISO-2), 2,3,23-trihydroxyolean-12-en-28-oic acid (arjunolic acid) and 2,19-dihydroxy-3-oxo-24-norolean-12-en-28-oic acid. Five flavonoid compounds exhibited higher levels in diseased samples, including 3,3′,4′,5-tetrahydroxy-7-methoxyflavone (rhamnetin), orientin-2″-O-xyloside, vaccarin, epicatechin-4′-O-β-d-glucopyranoside and genistein-8-C-apiosyl (1 → 6) glucoside. Additionally, six significantly different metabolites were annotated in the flavonoid and flavone metabolic pathways, including apigenin-6-C-glucoside (isovitexin), vitexin-2″-O-rhamnoside, kaempferol-3-O-glucoside (astragalin), apigenin-8-C-glucoside (vitexin), quercetin-3-O-glucoside (isoquercitrin) and luteolin-7-O-glucuronide. These significant triterpene and flavonoid metabolites were used for subsequent correlation analysis to explore the relationships between metabolites and microorganisms. Consistently, the overall classification heatmap of healthy and diseased samples (Figure 4E) illustrated that the levels of triterpene and flavonoid DAMs were generally higher in diseased samples. This indicates that root rot affects the composition and classification of root metabolites, particularly triterpene and flavonoid compounds. We then assigned these DAMs from healthy and diseased group comparisons to KEGG pathways to elucidate their biological functions. As presented in Figure 4D, KEGG enrichment analysis revealed that the pathways exhibiting the highest enrichment levels and significant differences were linoleic acid metabolism, phenylpropanoid metabolism, plant hormone signal transduction, sphingolipid metabolism and flavonoid and flavonol biosynthesis.

These data suggest that root rot can induce *P. tunicoides* roots to produce more triterpenes and flavonoids, potentially helping the plant resist pathogen invasion and maintain normal growth. Combining these findings with the transcriptomics data, we speculate that the resistance of *P. tunicoides* to root rot might be related to triterpene and flavonoid metabolism, with plant resistance enhanced through the accumulation of these compounds to prevent further pathogen invasion.

### 3.4 Microbial community diversity, composition and function analysis

Raw data were obtained through overlapping paired-end reads following sequencing. High-quality clean data were then acquired through quality control and chimera filtering. To investigate bacterial and fungal communities in *P. tunicoides* root and rhizosphere soil, we compared microbial diversity between samples from healthy and diseased specimens. This comparison included richness indices (Chao1), diversity indices (Shannon and Simpson) and OTU counts. Although not statistically significant, healthy *P. tunicoides* samples generally exhibited higher microbial richness and diversity than diseased samples in both rhizosphere soil and root (Supplementary Figure S1).

**Figure 5:**
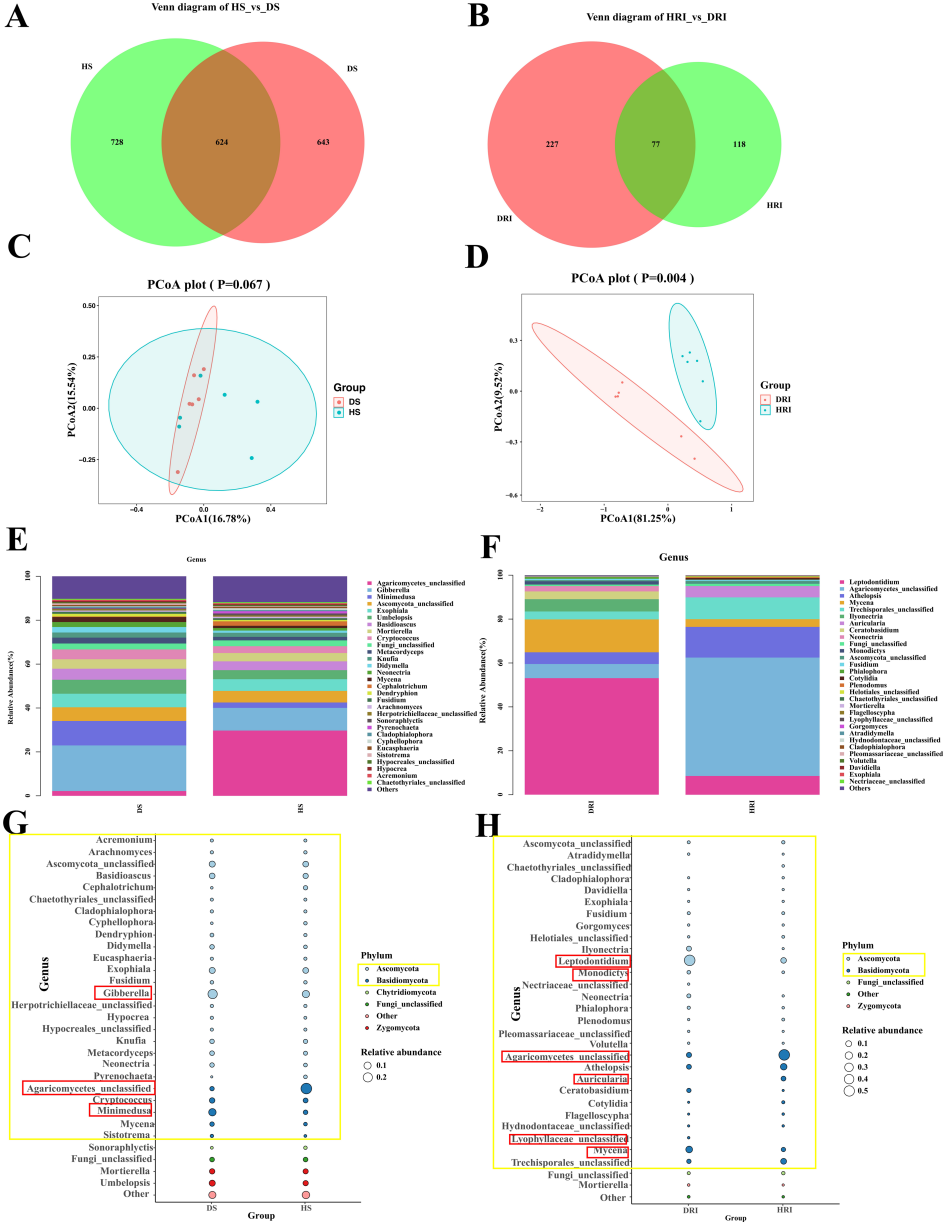
Comparative analysis of rhizosphere soil and root endophytic fungal communities in healthy and diseased *P. tunicoides*. **(A)** Rhizosphere soil and **(B)** root endophytic fungal OTU Venn diagrams. **(C)** Rhizosphere soil and **(D)** root endophytic fungal community PCoA plots. **(E)** Rhizosphere soil and **(F)** root endophytic fungal genus abundance group stacked bar charts. **(G)** Rhizosphere soil and **(H)** root endophytic fungal bubble plots depicting species annotation and relative abundance differences at the phylum and genus levels (yellow annotations indicate major differences at the phylum level, and red annotations indicate major differences at the genus level).

To elucidate microbial changes following root rot development in *P. tunicoides*, we employed linear discriminant analysis effect size and indicator analysis. Using the Kruskal–Wallis test (*p* < 0.05), we identified biomarkers distinguishing healthy and diseased *P. tunicoides* rhizosphere soil and root Analysis of variance (*p* < 0.05) revealed significant differences in the relative abundance of these biomarkers between healthy and diseased samples. We initially focused on fungal communities in rhizosphere soil and root. Venn diagram analysis (Figures 5A,B) demonstrated that root rot reduced fungal community abundance, particularly endophytic fungi (only 64% of the HR endophytic fungal abundance). PCoA was performed on the basis of differential OTU composition across all samples (Figures 5C,D). In rhizosphere soil, PCoA1 and PCoA2 explained 16.78 and 15.54% of the fungal community variation, respectively. However, the variation was not significant, as HSI and DSI did not cluster separately (Figure 5C). Conversely, for endophytic fungi, PCoA1 and PCoA2 explained 81.25 and 9.52% of the community variation, respectively. HRI and DRI formed distinct clusters, indicating significant fungal community variation (*p* = 0.004, Figure 5D). Comparisons of healthy and diseased rhizosphere soil and endophytic fungal community compositions revealed distinct differences in taxon abundance at the phylum level (Figures 5G–H), primarily in *Ascomycota* and *basidiomycota* (yellow annotations in Figure 5H). At lower taxonomic levels, rhizosphere soil fungal community analysis (Supplementary Table S6) uncovered a greater abundance of genera such as *Gibberella*, *Minimedusa*, *Fusarium, Mycena*, and *Umbelopsis* in DSI. Conversely, genera such as unclassified *Agaricomycetes*, *Cephalotrichum*, *Cyphellophora*, and *Pyrenochaeta* were more prevalent in HSI (Figure 5G). Endophytic fungal community analysis (Supplementary Table S7) revealed a higher abundance of *Leptodontidium*, *Mycena*, unclassified *Lyophyllaceae* and *Ilyonectria* in DRI, whereas unclassified *Agaricomycetes*, *Auricularia*, *Athelopsis* and unclassified *Trechisporales* were more prevalent in HRI (Figure 5H). These differences are clearly illustrated in the genus abundance group stacked bar charts (Figures 5E,F).

**Figure 6:**
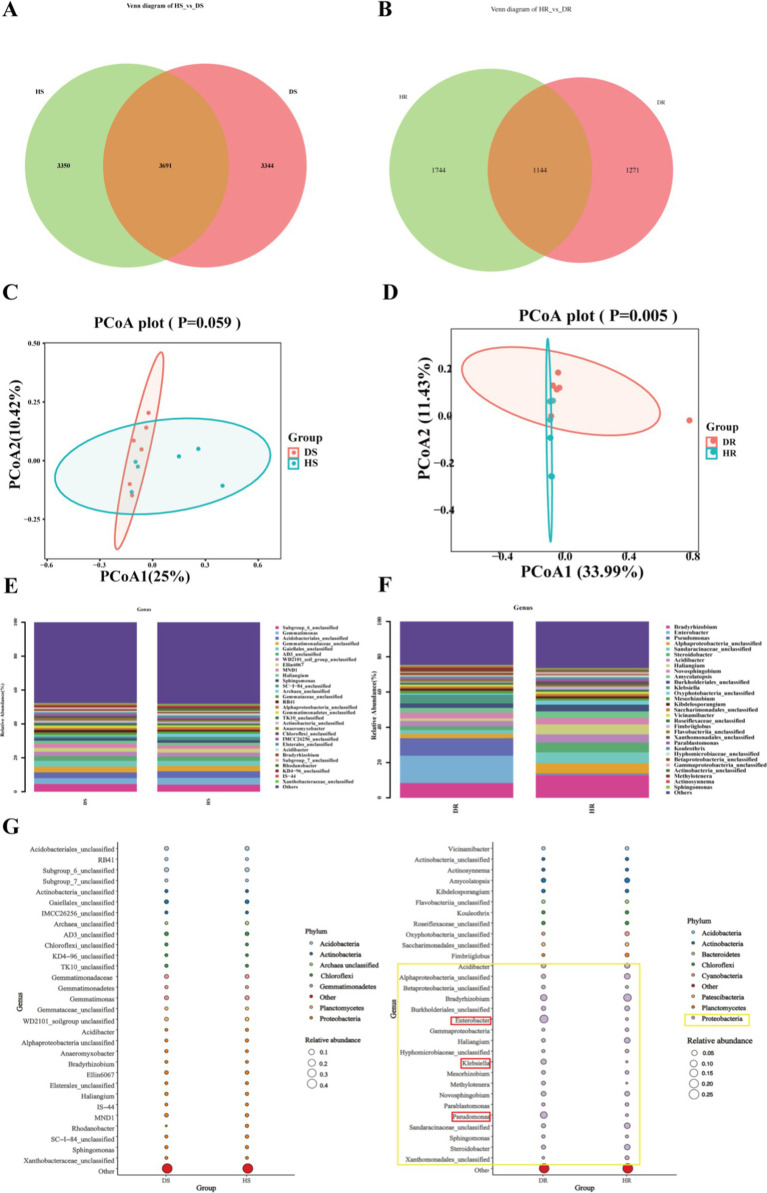
Comparative analysis of rhizosphere soil and root endophytic bacterial communities in healthy and diseased *P. tunicoides*. **(A)** Rhizosphere soil and **(B)** root endophytic bacterial OTU Venn diagrams. **(C)** Rhizosphere soil and **(D)** root endophytic bacterial community PCoA plots. **(E)** Rhizosphere soil and **(F)** root endophytic bacterial genus abundance group stacked bar charts. **(G)** Rhizosphere soil and **(H)** root endophytic bacterial bubble plots depicting species annotation and relative abundance differences at phylum and genus levels (yellow annotations indicate major differences at the phylum level, and red annotations indicate major differences at the genus level).

We conducted a comprehensive bacterial community analysis to examine the alterations in rhizosphere soil and endophytic bacteria of root following the onset of root rot in *P. tunicoides*. The Venn diagram (Figures 6A,B) revealed that root rot led to a reduction in bacterial community abundance, with a particularly pronounced effect on endophytic bacterial abundance. Notably, the abundance of endophytic bacteria in DR was only 83.6% of that observed in HR. We performed PCoA based on the differential OTU composition across all samples (Figures 6C,D). In rhizosphere soil, PCoA1 and PCoA2 accounted for 25 and 10.42% of the bacterial community variation, respectively. For endophytic bacteria, PCoA1 and PCoA2 explained 33.99 and 11.43% of the variation, respectively. However, the clustering between HR and DR samples did not exhibit clear separation. Our analysis of bacterial community composition (Figures 6E,F) identified the predominant bacterial phyla in rhizosphere soil, including *Proteobacteria, Actinobacteria, Acidobacteria, Chloroflexi, Gemmatimonadetes, Planctomycetes, Bacteroidetes, Verrucomicrobia, Firmicutes*, and *unclassified Archaea*. Interestingly, HS and DS rhizosphere soil communities displayed similar compositions at both the phylum and genus levels (Figure 6G). Of particular note was the significant enrichment of numerous Actinobacteria microorganisms in DS rhizosphere soil at the genus level (Supplementary Tables S8, S9). These included genera such as *Oryzihumus*, unclassified *Actinobacteria*, unclassified *Frankiales*, *Saccharopolyspora*, *Iamia*, *Geodermatophilus*, *Jatrophihabitans*, and *Kibdelosporangium*. By contrast, our comparative analysis of endophytic bacterial communities revealed marked differences in the abundance of specific taxa at the phylum level (Figure 6H). The most prominent differences between HR and DR endophytic bacteria were observed within Proteobacteria (highlighted in yellow in Figure 6H). At the genus level (Supplementary Table S10), we observed significant increases in the abundance of Proteobacteria genera such as *Pseudomonas*, *Enterobacter* and *Klebsiella* in DR samples compared with the findings in HR samples (highlighted in red in Figure 6H).

**Figure 7:**
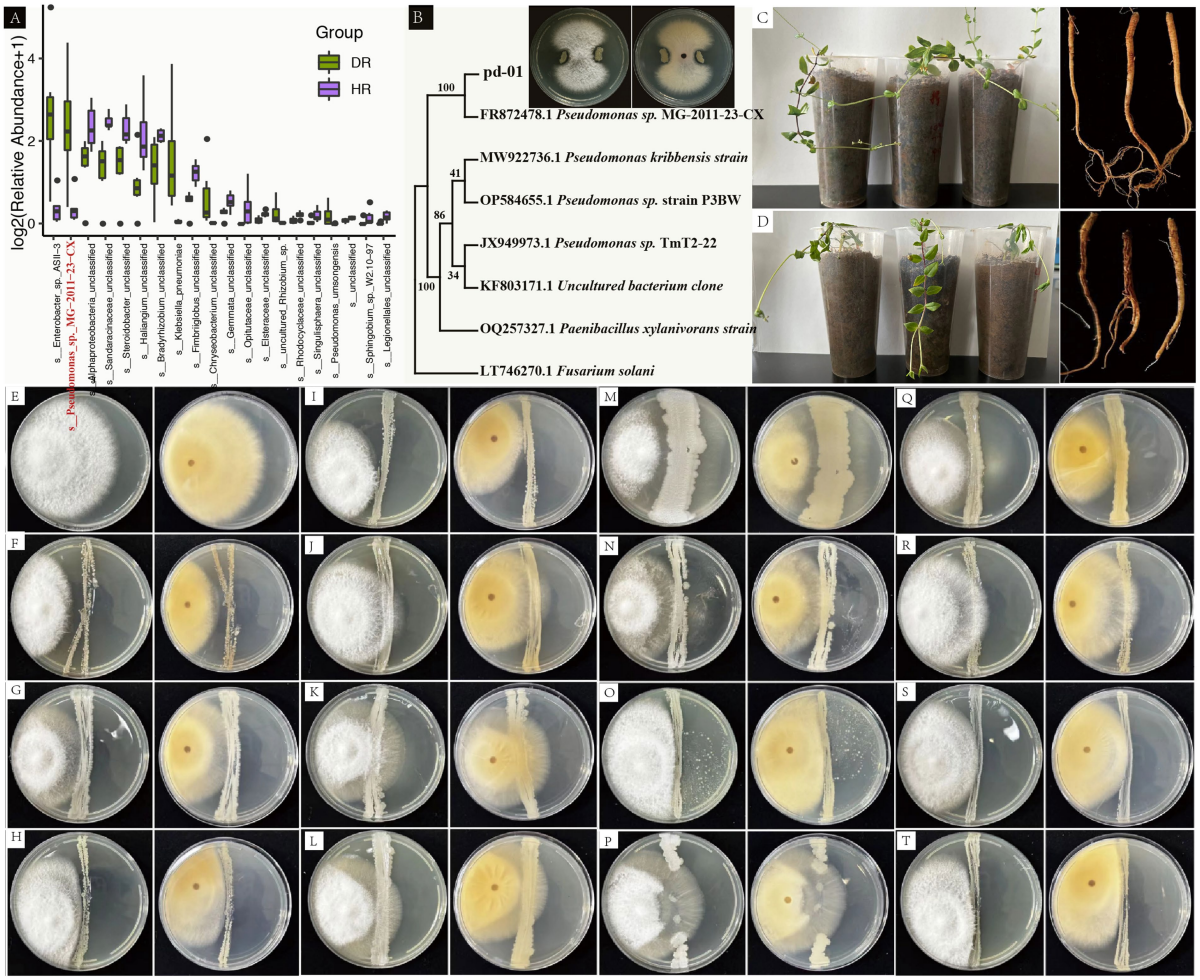
Pathogenicity assessment of *F. oxysporum* PF-01, isolation and identification of beneficial rhizosphere soil bacteria and plate antagonism experiments. **(A)** Species-level differences between HR and DR based on 16S rRNA gene amplicon sequencing (*Pseudomonas_*sp.*_*MG-2011-23-CX highlighted in red). **(B)** Phylogenetic tree analysis shows that pd-01 clusters with MG-2011-23-CX from **(A)** with 100% confidence. Plate antagonism assay indicates that pd-01 exhibits antagonistic activity against PF-01. **(C)** Potted plant condition and root morphology 30 days post-inoculation with sterile water (CK). **(D)** Potted plant condition and root rot morphology 30 days post-pathogen inoculation. **(E)** Control with *F. oxysporum* inoculation only. **(F–T)** Antagonistic activity of bacterial isolates against *F. oxysporum* (**F**: *Bacillus thuringiensis*, **G**: *Arthrobacter sp*., etc.).

To elucidate the etiology of root rot in *P. tunicoides* and identify potential antagonistic microorganisms, we isolated and characterized pathogenic fungi from diseased *P. tunicoides* samples. A fungal strain, designated PF-01, was isolated from DR. Through a combination of morphological observations, fungal rDNA-ITS and TEF-1α sequence analyzes and phylogenetic tree construction, PF-01 was conclusively identified as *Fusarium oxysporum*. We then validated the pathogenicity of this strain using Koch’s postulates, which demonstrated its ability to induce root rot in *P. tunicoides* with a 100% incidence rate (Figures 7C,D). based on these findings, we concluded that *F. oxysporum* is a primary pathogen responsible for root rot in *P. tunicoides*. In parallel, we sought to isolate potential beneficial microorganisms. We successfully isolated and identified more than 220 bacterial strains from the rhizosphere soil of diseased samples using a combination of microscopic observation, culture medium screening and gene sequencing techniques. These strains were classified into various genera, including *Bacillus* (e.g., *B. thuringiensis*, *B. subtilis*, *B. licheniformis*, *B. hominis*), *Arthrobacter* (e.g., *Arthrobacter* sp., *A. globiformis*), *Streptomyces* (e.g., *S. vinaceus*, *Streptomyces* sp.), *Cupriavidus* (e.g., *C. campinensis*, *Cupriavidus* sp.), *Paenibacillus*, *Pseudomonas* and *Paenarthrobacter*. Plate antagonism experiments revealed that these isolated strains could inhibit the growth of PF-01, producing variable inhibition zones (Figures 7E–T).

Notably, 16S rRNA gene amplicon sequencing identified a bacterial strain, named *Pseudomonas*_sp._MG-2011-23-CX, as the most significantly different at the species level, indicating its substantial enrichment in DR endophytic bacteria (Figure 7A). To investigate the potential role of *Pseudomonas* sp. in suppressing root rot, we isolated a *Pseudomonas* strain from *P. tunicoides* roots, which was designated pd-01. Interestingly, phylogenetic analysis revealed that pd-01 clustered with *Pseudomonas_sp.*_MG-2011-23-CX with 100% confidence (Figure 7B), suggesting they are likely the same strain. Subsequent plate antagonism tests demonstrated that pd-01 exhibited a clear inhibition zone against *F. oxysporum*, inhibiting fungal mycelial growth on PDA with an inhibition rate of 75% (Supplementary Table S11; Figure 7B).

### 3.5 Correlations among secondary metabolites, gene expression and microbial communities in *Psammosilene tunicoides*

**Figure 8:**
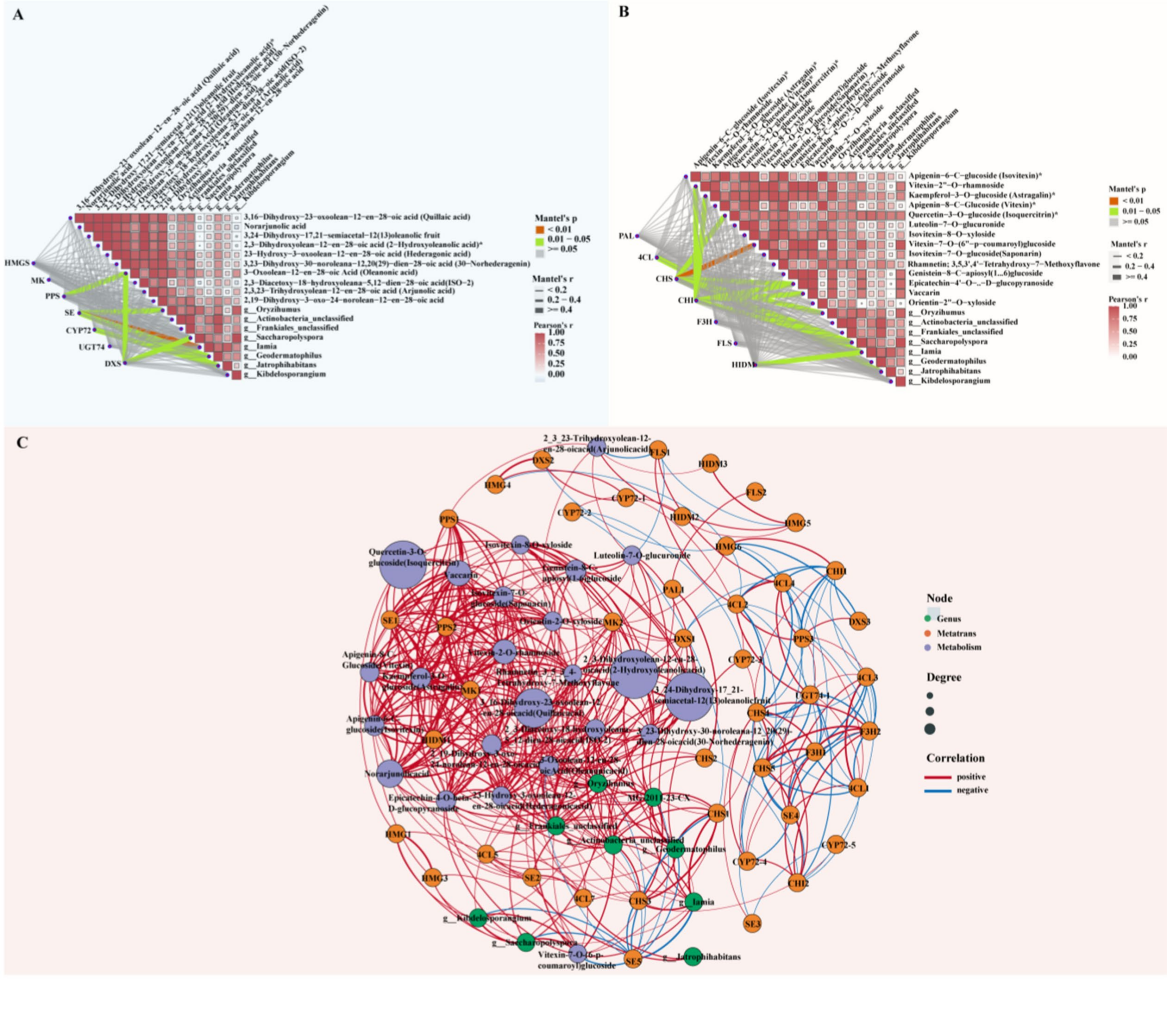
Asymmetric correlation heatmap and co-linearity analysis of differential metabolites, key genes and rhizosphere microorganisms. **(A)** Asymmetric correlation heatmap of triterpene compounds, key genes and beneficial bacteria in rhizosphere soil. **(B)** Asymmetric correlation heatmap of flavonoid compounds, key genes and beneficial bacteria in rhizosphere soil. Mantel’s *p* < 0.01: highly significant correlation (red); Mantel’s *p* = 0.01–0.05: significant correlation (green), Mantel’s *p* > 0.05: non-significant correlation (gray), Mantel’s *r* < 0.2: weak correlation (thin gray line), Mantel’s *r* = 0.2–0.4: moderate correlation (medium gray line), Mantel’s *r* > 0.4: strong correlation (thick gray line). **(C)** Co-linearity analysis among differential metabolites, key genes and rhizosphere microorganisms (green dots: significantly enriched actinomycetes in soil; orange dots: key enzymes for triterpene and flavonoid synthesis; purple dots: differential metabolites of triterpenes and flavonoids. Red lines indicate positive correlations, and blue lines indicate negative correlations).

We selected plant growth-promoting rhizobacteria (PGPR) that were significantly enriched in diseased rhizosphere soil and that are known to promote plant growth. We then analyzed the correlations of these bacteria with triterpenes and flavonoids with significantly different levels in diseased roots, in addition to key regulatory genes (Supplementary Table S12). First, in the correlation analysis of triterpene compounds, key genes in the triterpene biosynthesis pathway and significantly different microorganisms (Figure 8A), *PPS* and *DXS* had significant positive correlations with quillaic acid (Mantel’s *p* < 0.05). This combination was particularly correlated with *Oryzihumus* and unclassified *Frankiales*. SE displayed a correlation with 30-norhederagenin and featured significant positive correlations with microorganisms such as *Iamia*, *Oryzihumus* and *Geodermatophilus* (Mantel’s *p* < 0.05). *CYP72* exhibited a significant correlation with 2,19-Dihydroxy-3-oxo-20-en-olean-12-en-28-oic acid (Noorhagenic acid) and a strong association with *Jatrophihabitans*. *DXS* featured a significant positive correlation with oleanonic acid, particularly in association with *Saccharopolyspora* (Mantel’s *p* < 0.05). We performed correlation analysis among flavonoid compounds, key enzymes in the flavonoid biosynthesis pathway and significantly different microorganisms in rhizosphere soil. The results (Figure 8B) revealed a significant positive correlation between *CHS* and vitexin-7-O-(6″-p-coumaroyl) glucoside (Mantel’s *p* < 0.01). Moreover, microorganisms such as *Oryzihumus*, *Saccharopolyspora* and *Kibdelosporangium* also displayed strong associations with this interacting pair. *CHI* exhibited significant positive correlations with epicatechin-4′-O-β-d-glucopyranoside and vaccarin (Mantel’s p < 0.01), and this combination was positively correlated with microorganisms such as unclassified *Actinobacteria* and unclassified *Frankiales*. *4CL* displayed a strong association with kaempferol-3-O-glucoside. Some microorganisms (e.g., *Geodermatophilus*, *Jatrophihabitans*) also exhibited strong correlations with this interacting pair. Additionally, *4CL* had a significant correlation with quercetin-3-O-glucoside, and *Jatrophihabitans* had a strong association with this combination. *HIDM* and vitexin-7-O-(6″-p-coumaroyl) glucoside exhibited a significant correlation, with *Iamia* and *Jatrophihabitans* displaying strong associations with this combination.

To more intuitively observe the correlations among triterpene and flavonoid compounds with significant differences in abundance between healthy and diseased samples, key genes in their metabolic pathways and beneficial rhizosphere bacteria after root rot stress, we performed co-occurrence network analysis (Figure 8C; Supplementary Table S13). The relative abundance of *Oryzihumus* was positively correlated (*p* < 0.05) with triterpene metabolites such as quillaic acid, 33,24-dihydroxy-17,21-semiacetal-12(13) oleanolic fruit, 2-hydroxyoleanolic acid, hederagonic acid, 30-norhederagenin, oleanonic acid, ISO-2 and 2,19-dihydroxy-3-oxo-24-norolean-12-en-28-oic acid. This combination also exhibited significant positive correlations (*p* < 0.05) with triterpene genes such as *MK, PPS2, SE*, and *DXS*. The endophytic *Pseudomonas* strain MG-2011-23-CX featured significant positive correlations (*p* < 0.05) with the triterpene metabolites *ISO-2*, quillaic acid, 3,24-dihydroxy-17,21-semiacetal-12(13) oleanolic fruit, 2-hydroxyoleanolic acid, hederagonic acid, 30-norhederagenin, 2,19-dihydroxy-3-oxo-24-norolean-12-en-28-oic acid, rhamnetin and oleanonic acid. The endophytic strain MG-2011-23-CX exhibited significant positive correlations (*p* < 0.05) with key genes such as *SE5*. *Geodermatophilus* displayed significant positive correlations (*p* < 0.05) with the accumulation of 3,24-dihydroxy-17_21-semiacetal-12(13) oleanolic fruit, 2-hydroxyoleanolic acid, 30-norhederagenin and ISO-2. *Geodermatophilus* also featured significant positive correlations (*p* < 0.05) with triterpene key genes such as *SE5* and *CYP72-3*. The relative abundance of *Frankiales* was positively correlated (*p* < 0.05) with the accumulation of flavonoid metabolites such as isovitexin, vitexin, isovitexin-8-O-xyloside, vitexin-7-O-(6-p-coumaroyl) glucoside, rhamnetin and epicatechin-4-O-beta-d-glucopyranoside. Simultaneously, *Frankiales* was positively correlated with key genes such as *CHS1, CHS3*, and *HIDM1*. *Actinobacteria* was positively correlated (*p* < 0.05) with the accumulation of the flavonoid metabolite rhamnetin. *Actinobacteria* was also positively correlated (*p* < 0.05) with flavonoid key genes such as *4CL, CHS1*, and *CHS3*. *Geodermatophilus* displayed significant positive correlations (*p* < 0.05) with rhamnetin and flavonoid key genes such as *4CL5, CHS1*, and *CHS3.*

## 4 Discussion

Root rot, a serious pathogenic fungal infection, poses significant threats to the *P. tunicoides* industry and quality, causing substantial losses and hindering development. Despite its importance, the potential mechanisms of the defense response to *P. tunicoides* root rot and the beneficial microorganisms involved remain poorly understood. This study employed multi-omics approaches, including metabolomic, transcriptomic, microbiomics, and culturomics analyzes, to comprehensively explore the molecular processes and beneficial microbial communities associated with tolerance to *P. tunicoides* root rot. The results indicated that root rot both triggers changes in root transcription and metabolite content and significantly affects the diversity, assembly and function of root and rhizosphere microbial communities.

### 4.1 Transcriptomic sequencing analysis of gene regulation differences in healthy and diseased *Psammosilene tunicoides*

A comparative transcriptomic study between healthy and diseased *P. tunicoides* was conducted to systematically analyze differences in gene expression and provide important insights into the molecular responses associated with the disease. GO enrichment analysis revealed the significant enrichment of DEGs in biological processes, cellular components and molecular functions. Notably, DEGs in diseased samples were highly enriched in processes related to redox processes, signal transduction and transcriptional regulation, which were previously demonstrated to be closely associated with plant responses to pathogenic stress. According to Tiwari et al. (2017), plant signal transduction pathways, including the jasmonic acid (*JA*), ethylene (*ET*), cytokinin, abscisic acid (*ABA*) and strigolactone signaling pathways, can regulate plant growth under biotic and abiotic stress, microbial attack and symbiosis. Most of the DEGs associated with these signaling pathways were up-regulated in the diseased group, including genes involved in *SA* signaling (e.g., *NPR1, TGA*), ET signaling (e.g., *ERF, EIN, ETR*) and *ABA* signal perception and transduction (e.g., *PYR, PYL, ABF*). Additionally, the mitogen-activated protein kinase (*MAPK*) signaling pathway, a plant hormone signaling pathway, interacts with various plant hormones to regulate growth, development and responses to environmental stress. Genes responsible for regulating transcription factors (*NAC* and *WRKY*) related to adversity and growth in the *MAPK* signaling pathway were significantly up-regulated in diseased samples (see Supplementary Figure S2; Supplementary Table S14).

KEGG pathway analysis provides valuable insights into the complex biological functions of genes. In diseased samples, KEGG results revealed significant enrichment in pathways including amino sugar and nucleotide sugar metabolism (map00520), cysteine and methionine metabolism (map00270) and starch and sucrose metabolism (map00500). These pathways have been extensively documented to play crucial roles in plant responses to pathogenic stress. For instance, Majumdar et al. (2022) found that sugar beets (*Beta vulgaris* L.) respond to pathogen infection by activating genes involved in multiple metabolic pathways, particularly those related to amino sugar and nucleotide sugar metabolism (e.g., *PG, PNL, CEL*). The up-regulation of these genes might be linked to host defense responses and pathogen virulence. Notably, amino sugar and nucleotide sugar metabolism play a pivotal role in sugar beet resistance to *Rhizoctonia solani* and *Leuconostoc mesenteroides*, enhancing plant defense responses. Liu et al. (2022) demonstrated that treating plants with 0.1 g L^−1^ methionine enhanced enzyme activities, gene expression and fruit lignin content, thereby improving resistance to jujube black spot rot without directly inhibiting *Alternaria alternata* growth. Similarly, Scholes et al. (1994) found that barley leaves infected with powdery mildew exhibited increased acid invertase activity, resulting in glucose, fructose and sucrose accumulation in leaves, primarily in mesophyll cells, which enhanced barley’s resistance to powdery mildew.

KEGG pathway enrichment analysis also revealed the significant involvement of secondary metabolic pathways. The top 20 enriched pathways included phenylpropanoid biosynthesis (map00940), flavonoid biosynthesis (map 00941), steroid biosynthesis (map00100), sesquiterpenoid and triterpenoid biosynthesis (map00909), carotenoid biosynthesis (map00906), and isoquinoline alkaloid biosynthesis (map00950). Many plant secondary metabolites play crucial roles in defenses against herbivores, pests and pathogens or serve as an “intermediate medium” to recruit growth-promoting to disease-resistant. For example, phenylpropanoid compounds participate in multiple defense-related functions, including the formation of preformed and induced physical and chemical barriers. These compounds also participate in local or systemic signaling and potentially induce the expression of defense-related genes. Given that the flavonoid biosynthesis pathway is downstream of phenylpropanoid biosynthesis (Yu et al., 2022), enrichment of the flavonoid biosynthesis pathway was anticipated. Carotenoids, which are yellow, orange-red or red pigments in higher plants and animals, have been reported to confer greater resistance to pathogen infection. Orsi et al. (2023) assessed fruit susceptibility to *B. cinerea* in Micro-Tom near isogenic lines with mutations altering carotenoid profiles. They found that the wound-inoculated fruit of the mutants Delta carotene and tangerine, which produce a wider variety of carotenoids rather than primarily accumulating trans-lycopene, were less susceptible to the pathogen. Interestingly, differences in susceptibility between the mutants were only observed in ripe fruit after carotenoid formation, and these differences were associated with attenuated damage from ROS. Han et al. (2018) discovered that the methanolic extract of *Corydalis ternata* suppressed plant diseases caused by *Puccinia triticina* and *Colletotrichum coccodes*. They isolated three isoquinoline alkaloids (dehydrocorydaline, stylopine, and corydaline) from *C. ternata* as anti-fungal substances. These active compounds also exhibited *in vivo* anti-fungal activity against *P. triticina* and *C. coccodes*.

### 4.2 Differential expression patterns of key enzyme genes in triterpene and flavonoid synthesis pathways in healthy and diseased *Psammosilene tunicoides*

It is widely recognized that plants synthesize a diverse array of secondary metabolites, which primarily protect against biotic and abiotic stresses. Phenylpropanoids, triterpenoids and flavonoids are widely distributed compounds within this category. Comparative analysis of gene expression between healthy and diseased *P. tunicoides* samples revealed higher expression of key enzyme genes in the triterpene biosynthesis pathway in diseased samples, including *HMGS, MK, SQS, SQE, CYP72A* and *UGT*. This suggests that plants activate these genes to increase triterpene compound accumulation as a defense mechanism against disease. Previous studies demonstrated a close relationship between triterpene gene up-regulation and plant disease resistance (Singh et al., 2017). For instance, Singh et al. (2015) found that *SQS* gene silencing led to the down-regulation of brassinosteroid-6-oxidase-2, pathogenesis-related genes and NPRs, thereby reducing plant tolerance to bacterial and fungal infections as well as insect feeding. Xu et al. (2023) discovered that lines with silenced *GbCYP72A1* genes exhibited significantly decreased resistance to *Verticillium* wilt. Transcriptome sequencing and pathway enrichment analyzes indicated that the *GbCYP72A1* genes primarily influenced disease resistance through plant hormone signal transduction, plant–pathogen interactions and *MAPK* signaling pathways. He et al. (2018) found that *Fusarium* head blight (*FHB*) is a devastating disease affecting wheat crops globally, leading to yield losses and the accumulation of mycotoxins. *UGTs* could contribute to detoxification and enhance *FHB* resistance by glycosylating *DON* into *DON*-3-glucoside in wheat. However, In our research, some triterpene-related genes, such as certain *GPPS* and *FPPS* genes, were down-regulated in diseased samples, suggesting that pathway regulation under pathogen stress might depend on specific plant requirements or growth stages.

Meanwhile, the key genes such as *PAL*, *4CL*, *CHI*, *C4H*, *ANS*, and *FLS* were up-regulated in diseased samples. The up-regulation of these crucial enzyme genes in the flavonoid compound synthesis pathway is expected to promote flavonoid accumulation, thereby enhancing plant resistance to disease. Wang et al. research (Wang et al., 2022) demonstrated a direct relationship between flavonoid gene up-regulation and plant disease resistance. For example, Xu et al. (2019) found that humic acid treatment in grapevines up-regulated *PAL*, *C4H*, and *4CL*, promoting the accumulation of phenylpropanoid metabolites and significantly reducing the severity of gray mold caused by *Botrytis cinerea*, despite humic acid exhibiting no anti-fungal activity *in vitro*. Alariqi et al. (2023) proposed that over-expression of *Gh4CL3* led to increased *4CL* activity, augmenting the levels of phenolic precursors (cinnamic, p-coumaric, and sinapic acids) and channeling them into lignin and flavonoid biosynthesis, thereby enhancing resistance to *V. dahliae*.

### 4.3 Accumulation patterns of triterpenoids and flavonoids compounds in healthy and diseased *Psammosilene tunicoides* plants

Our metabolomic data analysis identified 295 differential metabolites across 10 major categories, including flavonoids, terpenoids, lipids, phenolic acids, lignans and coumarins. These metabolites play crucial roles in various plant physiological functions, particularly in response to environmental stresses and diseases. Further analysis of DAMs revealed that the levels of 56 metabolites were elevated, and those of three metabolites were reduced in diseased *P. tunicoides*. Plants can activate entire or partial defense networks, with different activation patterns observed under stress conditions. In our study, pathogen infection in *P. tunicoides* induced a greater accumulation of triterpenoids and flavonoids. Among the top 20 DAMs, 10 were triterpenoid compounds, including 2-aldehydo-A (1)-norlup-20(29)-en-27,28-dioic acid (zizyberanal acid), norarjunolic acid and 2,3,19-trihydroxyurs-12-en-23-formyl-28-oic acid. Five were flavonoid compounds, such as rhamnetin, orientin-2″-O-xyloside, vaccarin, epicatechin-4′-O-β- d-glucopyranoside and genistein-8-C-apiosyl (1 → 6) glucoside. The levels of these compounds were significantly increased in the diseased group (highlighted in Figures 3D,E), consistent with the KEGG enrichment analysis results from the transcriptome analysis (Figure 2D).

Plant-derived secondary metabolites play crucial roles in facilitating environmental adaptation. These compounds function to deter herbivores and natural predators, defend against pathogenic microorganisms, assist in adapting to alterations in the physical and chemical environment, and mediate both intra-and interspecific interactions among plants. Hirai et al. (2000) identified three antimicrobial triterpenoids—euscaphic, tormentic and myrianthic acids—from unripe strawberry fruits that were wounded and inoculated with *Colletotrichum musaeconidia*. These triterpenoids exhibited antimicrobial activity against *C. musae*, and they potentially contribute to resistance against the strawberry root pathogen *Phytophthora fragariae* var. *fragariae*. Azizah et al. (2020) found that rhamnetin, a flavonoid glycoside, exhibited antimicrobial activity against *B. cereus*, *Staphylococcus aureus* and *P. aeruginosa*, with inhibition zones of 9, 9, and 13 mm, respectively. Furthermore, rhamnetin is considered to have anti-fungal activity in plants, and it might have a significant role in the antimicrobial properties of *V. denticulata*. In our research, the DAMs involved in triterpenoid and flavonoid biosynthesis were greater accumulation in diseased plant, indicating their potential direct involvement in regulating plant immune defense mechanisms.

### 4.4 Impact of disease on the composition and function of the *Psammosilene tunicoides* root and rhizosphere microbial flora

The rhizosphere microbial community has a crucial role in plant defense against soil-borne pathogens, with healthy microbial communities typically exhibiting higher diversity and functional redundancy, thereby contributing to enhanced plant disease resistance (Cook et al., 1995). This study demonstrated that root rot affects both gene transcription and metabolism in *P. tunicoides* and significantly impacts the root endosphere and rhizosphere microbial communities. The high-throughput sequencing analysis revealed significant changes in the root endosphere and rhizosphere microbial communities of diseased plants compared with those in healthy plants. The α-diversity of these microbial communities was significantly higher in healthy plants than in diseased plants, with the lower diversity potentially weakening the plant’s disease resistance. The 16S rDNA V3–V4 amplicon sequencing revealed significant enrichment of mostly beneficial growth-promoting bacterial groups in DR endophytes. These include genera such as *Enterobacter*, *Pseudomonas*, *Klebsiella*, *Chryseobacterium*, *Stenotrophomonas*, *bacillus, Rhizobium*, and *Massilia*, which have been reported to have good inhibitory effects on soil-borne pathogens. Fatima and Anjum (2017) explored the potential of *Pseudomonas aeruginosa PM12* to induce systemic resistance in tomatoes against *FWD*. After purifying bacterial crude extracts and analyzing them using gas chromatography–MS, three compounds were identified: 3-hydroxy-5-methoxybenzyl alcohol (*HMB*), eugenol and tyrosine. *HMB*, when used as a soil drench, significantly improved resistance to *FWD* in tomatoes. Gong et al. (2019) reported that Vt-7 (identified as *Enterobacter asburiae*) could completely inhibit the growth of *Aspergillus flavus* and seven other important fungal pathogens through the production of volatiles. Additionally, Vt-7 significantly inhibited *A. flavus* infection in stored peanuts and reduced the expression of aflatoxin biosynthesis-related genes, thus preventing aflatoxin production. Scanning electron microscopy further confirmed that Vt-7 volatiles could prevent *A. flavus* spore germination on peanut surfaces and severely damage spore structures. Gao et al. (2021) conducted a comprehensive analysis of bacterial and fungal communities across 12 parts of chili pepper (*Capsicum annuum L.*) and investigated the effects of FWD on plant microbiome assembly, symbiotic patterns and ecological functions. Their findings revealed that bacteria such as *Enterobacter*, *Klebsiella*, *Citrobacter* and *Pseudomonas* sp. play pivotal roles in the plant microbiome, promoting plant growth and enhancing disease resistance. These bacteria improve plant health by recruiting beneficial microorganisms that suppress soil pathogens. Berendsen et al. (2018) demonstrated that *Arabidopsis thaliana*, following leaf defense activation, promoted rhizosphere colonization by three bacteria, including *Stenotrophomonas*. These bacteria synergistically formed biofilms *in vitro*, and their combined action enhanced the plant’s systemic resistance to downy mildew while promoting growth. In a study by Kang et al. (2020), *Typha angustifolia* significantly increased the variety of rhizobia (*Rhizobium*) in the rhizosphere, indicating its contribution to nitrogen fixation. Conversely, *Bothriochloa ischaemum* enriched bacteria such as *Massilia*. These plants both support rich soil microbial communities and potentially participate in phytoremediation by increasing soil nitrogen content and acting as bioaccumulators for heavy metals (Pb and Mn).

Consistent with the discovery of beneficial bacteria in diseased roots, we observed significant enrichment of numerous actinobacteria in the rhizosphere soil of diseased plants, including *Oryzihumus*, unclassified *Actinobacteria*, unclassified *Frankiales*, *Saccharopolyspora*, *Iamia*, *Geodermatophilus*, *Jatrophihabitans*, and *Kibdelosporangium*. This underscores the critical role of actinobacteria in plant health management. In addition, there is also an enrichment of Bacillus species. *Actinobacteria* species are well-known potential biocontrol agents for various plant diseases because of their ability to produce broad-spectrum antibiotics. They participate in plant–pathogen interactions through multiple biological mechanisms, including direct pathogen growth inhibition, rhizosphere environment improvement and the promotion of plant growth and nutrient uptake (Cuesta et al., 2012). Qi et al. (2022) demonstrated that inoculation with *Frankia casuarinae* CcI3 (*Frankia* F1) significantly enhanced ginseng plant growth and inhibited the growth of the pathogen *F. solani*. The inoculation also improved soil pH, nutrient content and enzyme activity, contributing to improved plant disease resistance and stress tolerance. Prabha et al. (2014) isolated 50 actinobacterial strains from tomato rhizosphere soil, among which *Saccharopolyspora erythraea* displayed significant antagonistic activity against *R. solani* and *Sclerotinia sclerotiorum*. This indicates the potential application of *Saccharopolyspora* in controlling stem rot and damping-off diseases in tomatoes. Shami et al. (2022) employed metagenomic whole-genome shotgun sequencing to analyze the rhizosphere microbial communities of *Dipterygium glaucum* and the surrounding soil microbiota response. Their study suggested that *Geodermatophilus* can influence root sampless through complex symbiotic relationships, thereby promoting plant resistance under adverse conditions. Barros et al. (2022) identified *Jatrophihabitans* as a potential target for the biological control of the pathogen *M. javanica*. Qin et al. (2015) identified *K. phytohabitans* KLBMP 1111 T as a plant growth-promoting endophytic actinobacterium isolated from the oilseed crop *Carica papaya* in a hot and dry valley. Its complete genome contains one chromosome with identified gene clusters related to natural product synthesis and plant growth promotion, such as cytokinin, ACC deaminase and siderophores, which are beneficial to plants. Duan et al. (2023) found that *Iamia* is associated with sugarcane disease resistance and is involved in improving plant growth and highlighted its denitrification activity. Their study demonstrated that *Iamia* interacts with other bacteria to promote mutual growth and plays a crucial role in resisting sugarcane smut disease.

To further identify potential beneficial microorganisms, we isolated and identified rhizosphere soil bacteria and conducted plate antagonism experiments. The results revealed that the isolated bacteria, including *B. thuringiensis*, *B. subtilis*, *B. licheniformis*, *B. hominis*, *Arthrobacter* sp., *Streptomyces*, *Cupriavidus*, *Paenibacillus*, *Pseudomonas*, and *Paenarthrobacter*, exhibited notable inhibitory effects against *F. oxysporum* (Figures 7E–T). These beneficial microorganisms have been extensively documented in the scientific literature, particularly concerning their roles in promoting plant growth through nitrogen fixation, phosphorus and potassium solubilization (Khan et al., 2022), being biocontrol agents (Ramlawi et al., 2021), producing exogenous anti-fungal metabolites (Yuan and Crawford, 1995), synthesizing phytohormones (Zhang et al., 2024), enhancing plant resistance to pathogens and inducing systemic resistance (Kim et al., 2016; Nguyen et al., 2023).

In recent years, researchers have emphasized the crucial role of the microbiome in plant disease resistance. *Pseudomonas* and *Fusarium* was present in both root endophytic and rhizosphere soil microbial communities. Through microbial amplicon sequencing, we isolated and identified the potential pathogen *F. oxysporum* PF-01 and the beneficial bacterium *Pseudomonas* pd-01. Interestingly, pd-01 clustered with the differential strain MG-2011-23-CX from the 16S sequencing results in the phylogenetic tree with 100% confidence, leading us to conclude that pd-01 and MG-2011-23-CX are the same strain. We verified that PF-01 can cause root rot in *P. tunicoides* using Koch’s postulates. In plate antagonism tests, *Pseudomonas* pd-01 inhibited the growth of *F. oxysporum* PF-01. It is well established that *Pseudomonas* has great potential to control various pathogenic fungi and bacteria through its production of broad-spectrum antibiotics. Haas and Keel (2003) reported that the biocontrol capacity of *Pseudomonas* primarily depends on active root colonization, the induction of plant systemic resistance and the production of diffusible or volatile anti-fungal antibiotics. Known antibiotics with biocontrol properties include phenazines, 2,4-diacetylphloroglucinol, pyrrolnitrin and hydrogen cyanide. Overall, our research results suggest that pd-01 has the potential for development as a biocontrol agent against *P. tunicoides* root rot.

### 4.5 Asymmetric correlation heatmap and co-occurrence network analyzes reveal “gene–metabolite–microbe” interactions

We screened for PGPR enriched in the rhizosphere soil of diseased samples. By combining the screened bacteria with significantly differentiated triterpenoids and flavonoids and their key genes, we conducted asymmetric correlation heatmap and co-occurrence network analyzes of metabolite–gene–microbe associations. Our results indicated significant correlations among several flavonoids, triterpenoids, key regulatory genes and beneficial microbial genera. For example, *CHS* had a significant positive correlation with vitexin-7-O-(6″-p-coumaroyl) glucoside (Mantel’s *p* < 0.01) and strong associations with microbial genera including *Oryzihumus* and *Saccharopolyspora*. This suggests that the biosynthesis of specific flavonoids is closely related to the activity of these beneficial rhizosphere microorganisms, potentially enhancing the plant’s ability to mitigate root rot. Similarly, *CHI* demonstrated strong correlations with epicatechin-4′-O-β-d-glucopyranoside and vaccarin. This combination’s association with microbes including unclassified *Actinobacteria* and unclassified *Frankiales* highlights the impact of flavonoid biosynthesis pathways on microbial communities. The relationship between triterpenoid biosynthesis and microbial communities is also significant. The *PPS* and *DXS* genes displayed positive correlations with quillaic acid and the microbial genera *Oryzihumus* and unclassified *Frankiales*, further supporting the role of beneficial microorganisms in regulating *P. tunicoides* triterpenoid accumulation. Additionally, the strong associations among the *CYP72* gene, Noorhagenic acid and the microbe *Jatrophihabitans* suggest potential regulatory roles of triterpenoid biosynthesis in specific microorganisms.

Building on these findings, we hypothesize that *P. tunicoides* may employ a coordinated defense strategy wherein root-secreted specialized metabolites selectively enrich rhizosphere-beneficial microbiota under pathogenic stress, culminating in integrated tolerance to concurrent biotic and abiotic challenges. Luo et al. (2021) discovered that ginsenosides, autotoxic substances secreted by the rhizosphere of *Panax notoginseng*, significantly enrich *Burkholderia*–*Caballeronia*–*Paraburkholderia* in the rhizosphere soil. They isolated eight *Burkholderia* strains from the rhizosphere soil of *P. notoginseng*. Antagonistic experiments revealed that the *Burkholderia* B36 strain could degrade ginseng autotoxic substances (Rb, Rg, and Rd1) and inhibit the growth of the rust rot pathogen *Ilyonectria destructans*, alleviating the negative plant–soil feedback phenomenon in *P. notoginseng*. Fujimatsu et al. (2020) found that soyasaponin Bb, a secondary metabolite secreted by the soybean (*Glycine max*) rhizosphere, significantly influenced the structure of soybean soil rhizosphere microbiota. In soil treated with soyasaponin Bb and soybean rhizosphere soil, soyasaponin Bb significantly enriched potentially growth-promoting and disease-resistant beneficial bacteria, including *Novosphingobium*, promoting soybean nitrogen absorption and inducing plant resistance system expression. Huang et al. (2019) discovered that plant-specific triterpenoid compounds play crucial roles in assembling and regulating plant rhizosphere microbiota. In studies on the model plant *Arabidopsis*, they found that its roots produce a series of special triterpenoid substances, guiding the establishment and maintenance of an *Arabidopsis* rhizosphere-specific microbiota. This enables the plant to shape and adjust the microbial communities within and around the rhizosphere according to its own needs. Our study similarly indicated that the accumulation of triterpenoids and flavonoids might play a key role in the defense against root rot in *P. tunicoides*. Singh et al. (2015) found that the melon transporter genes *CmMATE1* and *ClMATE1* participate in the transport of cucurbitacin B (*CuB*) and CuE. These genes can selectively transport *CuB* and *CuE* from roots to the rhizosphere. *CuB*, serving as a carbon source, selectively recruits large numbers of *Enterobacter* and *Bacillus* specimens, thereby altering the rhizosphere microbial community structure and strongly inhibiting the *Fusarium* wilt disease (FWD) pathogen (*F. oxysporum*). This represents an efficient defense system against soil-borne *Fusarium* in melon.

However, this study focused on specific metabolic pathways, including flavonoid and triterpenoid biosynthesis pathways, but plant immune responses are a complex process involving multiple pathways. Future research should employ more comprehensive metabolomics approaches to explore other unaddressed metabolic pathways and their contributions to disease resistance.

## 5 Conclusion

This study examined healthy and diseased *P. tunicoides* roots and rhizosphere soil. Using transcriptomics, metabolomics and microbial sequencing to analyze changes in *P. tunicoides* after root rot occurrence, our results illustrated that flavonoid and triterpenoid metabolism actively dominates *P. tunicoides* resistance to root rot. We mined plant transcriptome, metabolome and microbiome resources, revealing that the expression of triterpenoid biosynthesis genes (e.g., *HMGS*, *DXS, SQS, CYP450*) and flavonoid biosynthesis genes (e.g., *PAL, CHS, CHI*) were up-regulated in diseased *P. tunicoides* compared with their expression in healthy specimens. Furthermore, we hypothesize that when *P. tunicoides* plants are stimulated by external adverse factors, their roots accumulate and secrete large amounts of specific triterpenoid and flavonoid secondary metabolites through gene regulation. These secondary metabolites can directly resist pathogen invasion while also enriching growth-promoting and disease-resistant beneficial bacterial communities. This includes the endophytic *Pseudomonas* strain MG-2011-23-CX and antagonistic bacteria isolated and identified from the soil, including *B. thuringiensis*, *B. subtilis*, *B. licheniformis*, *B. hominis*, *Arthrobacter* sp., *Streptomyces*, *Cupriavidus*, *Paenibacillus*, *Pseudomonas*, and *Paenarthrobacter.* as well as a series of actinomycetes enriched in the rhizosphere soil, including *Oryzihumus*, unclassified *Actinobacteria*, unclassified *Frankiales*, *Saccharopolyspora*, *Iamia*, *Geodermatophilus*, *Jatrophihabitans*, and *Kibdelosporangium*. Most of these actinomycetes exhibited positive correlations with triterpenoid and flavonoid compounds and their key genes. This study provides new insights and potential applications for the future ecological cultivation and management of *P. tunicoides*.

## Data Availability

The datasets presented in this study can be found in online repositories. The names of the repository/repositories and accession number(s) can be found at: https://www.ncbi.nlm.nih.gov/, PRJNA1197671, BioProject ID PRJNA1197678, BioProject ID PRJNA1197714, PRJNA1197702, PRJNA1204204.

## Footnotes

^1^ http://www.r-project.org

^2^ https://www.genome.jp/kegg/

^3^ http://www.kegg.jp/kegg/compound/

^4^ http://www.kegg.jp/kegg/pathway.html
