# Supplementary figures and images for: Integrated analysis of transcriptomics and metabolomics and high-throughput amplicon sequencing reveals the synergistic effects of secondary metabolites and rhizosphere microbiota on root rot resistance in Psammosilene tunicoides
Source: Front Microbiol. 2025 Apr 14;16:1554406. doi: 10.3389/fmicb.2025.1554406 (PMC12034638; doi:10.3389/fmicb.2025.1554406)

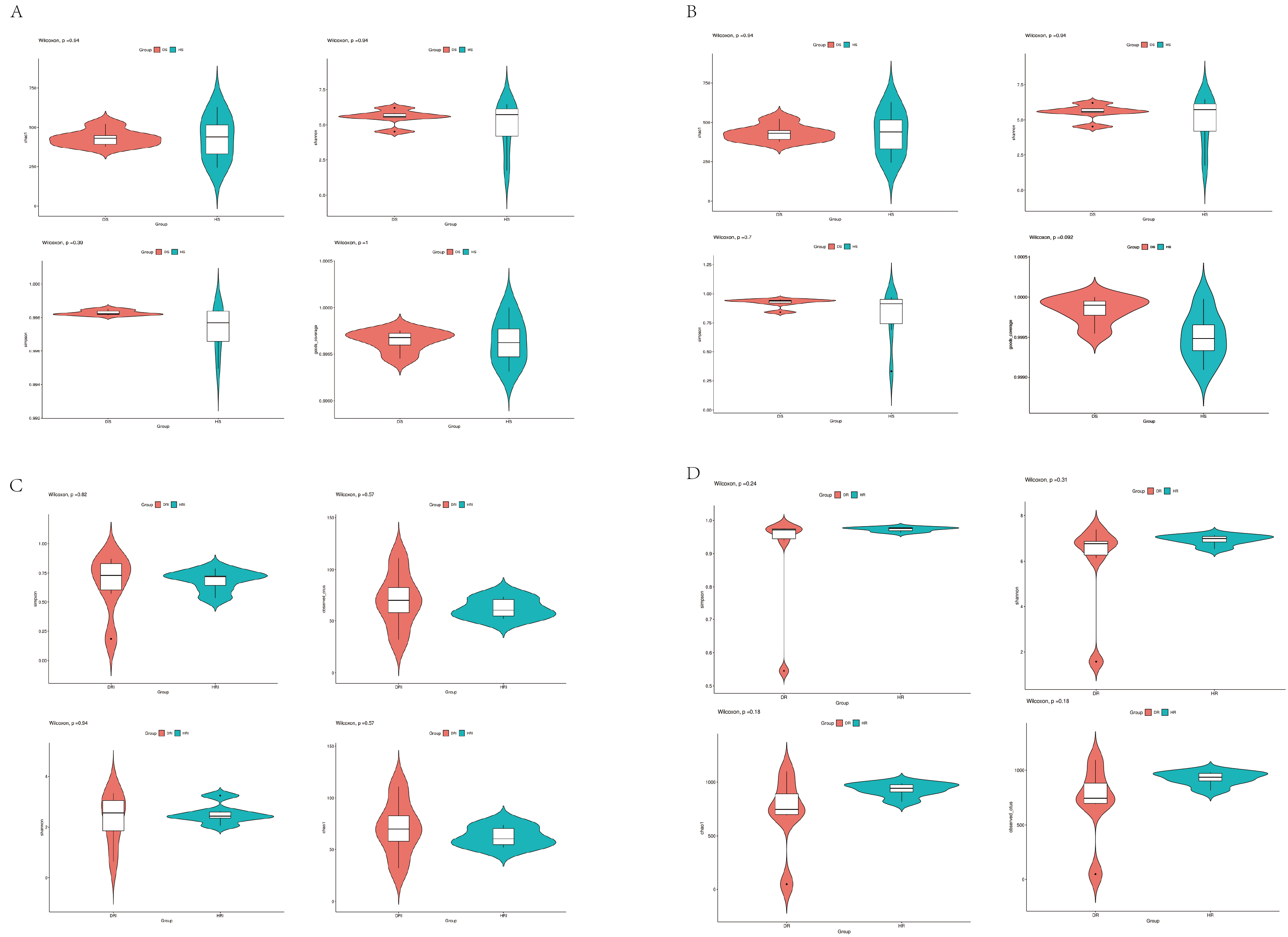

Supplement: Supplementary file 15 [file Image_1.png]

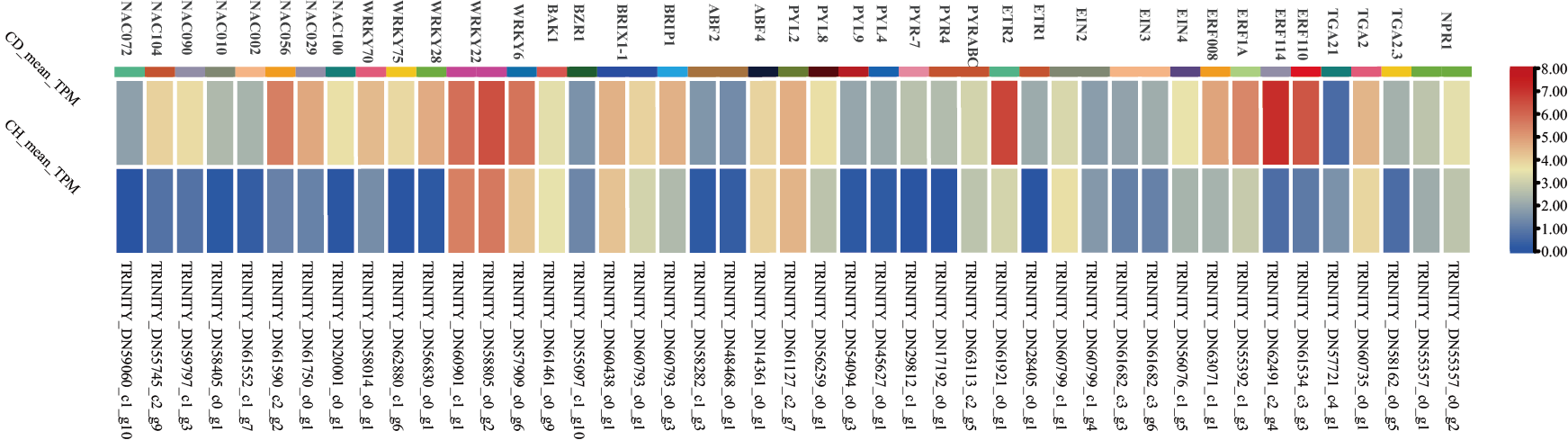

Supplement: Supplementary file 16 [file Image_2.png]
